# Paired Tissue and Plasma Comprehensive Genomic Profiling in Advanced Solid Tumors: A Retrospective Study from India

**DOI:** 10.3390/curroncol33100575

**Published:** 2026-09-25

**Authors:** Ankur Bahl, Nitesh Rohatgi, Suman S. Karanth, Aakriti Aggarwal, Rohit Bharat, Nippun Sandhir

**Affiliations:** 1 Medical Oncology Department, Fortis Memorial Research Institute, Gurugram 122003, India; ankur.bahl@fortishealthcare.com (A.B.); nitesh.rohatgi@fortishealthcare.com (N.R.); suman.karanth@fortishealthcare.com (S.S.K.); aakritiaggarwal2496@gmail.com (A.A.); 2 Department of Medical Affairs, Guardant Health India Pvt. Ltd., Hyderabad 500032, India; 3 Department of Medical Affairs, Guardant Health Pte Ltd., Singapore 138567, Singapore; nsandhir@guardanthealth.com

**Keywords:** advanced solid tumors, comprehensive genomic profiling, circulating tumor DNA, tissue-based genomic profiling, paired tissue–plasma testing, next-generation sequencing, precision oncology, India

## Abstract

Background: Tissue and plasma comprehensive genomic profiling (CGP) sample different compartments and may yield nonoverlapping clinically relevant findings. We assessed the added patient-level yield of paired testing in a pan-cancer cohort from India. Methods: This retrospective, single-institution study included patients with stage IV solid tumors who underwent paired tissue and plasma CGP between November 2022 and October 2024. The primary endpoint was detection in a patient of an OncoKB Level 1 or Level 2 or R1 alteration, high tumor mutational burden, or microsatellite instability. Molecularly informed therapy and laboratory turnaround time were assessed descriptively. Results: Of 158 patients, 123 matched evaluable pairs were analyzed. Either modality detected a clinically relevant finding in 46 of 123 patients (37.4%; 95% confidence interval, 29.4–46.2%): tissue CGP in 33 (26.8%), plasma CGP in 35 (28.5%), both in 22 (17.9%), tissue only in 11 (8.9%) and plasma only in 13 (10.6%). This complementarity remained bidirectional across exploratory analyses, including a subgroup that excluded non-small cell lung cancer (33.0%), all 139 technically successful pairs (33.8%), all 158 paired sample sets (32.3%), and even when the endpoint was restricted to OncoKB alterations (22.0%). Among 91 patients with therapy-alignment data, 25 (27.5%) received molecularly informed therapy. Median laboratory turnaround time was 7 days for plasma and 13 days for tissue CGP. Conclusions: Tissue and plasma CGP were complementary rather than interchangeable in this selected cohort: either modality alone would have missed clinically relevant findings. Whether this improves outcomes is untested; prospective studies linking testing strategy to treatment delivery and survival are needed.

## Simple Summary

Genomic testing of tumor tissue guides treatment for many advanced cancers, and blood-based testing is increasingly used alongside it. Most of that evidence comes from lung cancer, and real-world data from India are limited. In this retrospective study of 123 selected patients with cancers that had spread, the two tests proved complementary rather than interchangeable: blood testing found treatment-relevant changes that tissue testing missed in 13 patients, and tissue testing found changes that blood missed in 11. Blood results also arrived sooner, and in patients whose tissue sample was inadequate for testing, blood was sometimes the only genomic information available. Paired testing may therefore help most where tissue is limited, or results are needed quickly. Because treatment outcomes were not analyzed, this study cannot show whether paired testing helps patients live longer or better; prospective studies are needed to answer that.

## 1. Introduction

Next-generation sequencing has become an important component of precision oncology for patients with advanced solid tumors. Across multiple tumor types, genomic profiling can identify alterations associated with response or resistance to targeted therapies, immunotherapy biomarkers, or eligibility for molecularly matched clinical trials [1,2,3,4]. Professional guidance supports broad molecular profiling in settings where actionable biomarkers are common and recommends assessment of tumor-agnostic biomarkers when matched treatment options are available [3,4].

Tumor tissue remains the foundational substrate for comprehensive genomic profiling because it permits direct assessment of malignant tissue in a histopathologic context. Tissue-based testing can be integrated with morphology, immunohistochemistry, tumor content assessment, and other pathology findings. However, tissue CGP has well-recognized limitations. Biopsy may be unsafe or impractical, available tissue may be inadequate or degraded, and a single archival or site-specific specimen may not capture the full molecular heterogeneity of metastatic disease. These limitations are particularly relevant when prior systemic therapy has selected resistant subclones or when treatment decisions must be made before repeat tissue acquisition is feasible [5,6,7,8].

Plasma circulating tumor DNA comprehensive genomic profiling (plasma ctDNA CGP; hereafter plasma CGP) provides a complementary blood-based approach to tumor genomic profiling. Plasma testing is minimally invasive, can often be completed more rapidly than tissue testing, and may reflect DNA shed from multiple disease sites [9,10,11,12,13,14,15]. At the same time, plasma CGP is not a substitute for tissue in all settings. Low tumor fraction, limited ctDNA shedding, disease distribution, and assay-specific factors can lead to false-negative plasma results, while clonal hematopoiesis and other non-tumor sources of cell-free DNA may complicate variant interpretation [16,17,18,19,20,21,22,23,24]. Thus, tissue and plasma CGP have different strengths, limitations, and biologic sampling frames.

These differences provide the rationale for concurrent or closely paired tissue and plasma profiling. Rather than viewing the two modalities as interchangeable, paired testing may increase detection of clinically relevant alterations by reducing the blind spots of either approach alone. The strongest clinical precedent for this strategy comes from advanced non-small cell lung cancer (NSCLC), where timely identification of targetable driver alterations is central to first-line treatment selection [25,26]. Evidence is also emerging in selected non-NSCLC tumor types, including metastatic colorectal cancer and breast cancer, but real-world pan-tumor paired-testing data remain limited [11,27,28,29]. Published experience from Asia is largely confined to a small number of single-country implementation cohorts [30,31], and data from India are especially sparse, despite increasing use of CGP in routine oncology practice.

We therefore performed a retrospective paired-sample study of tissue and plasma CGP in patients with stage IV solid tumors treated at a tertiary cancer center in India. The primary aim was to evaluate the added patient-level yield and complementarity of the two modalities for detecting clinically relevant genomic findings. We also assessed assay turnaround time and described how tissue and plasma findings contributed to molecularly informed treatment decisions.

## 2. Materials and Methods

### 2.1. Study Design and Population

This retrospective, single-institution observational study included patients with stage IV solid tumors who underwent comprehensive genomic profiling (CGP) of both tissue and plasma during routine clinical care from November 2022 to October 2024. Reporting followed STROBE guidelines [32].

Eligible patients had both tissue and plasma CGP reports; plasma-only and tissue-only cases were excluded. The primary analytic cohort comprised matched evaluable tissue–plasma pairs after two sequential exclusions, which are conceptually distinct. First, sample sets in which either assay was technically unsuccessful were excluded; all 19 such failures were tissue samples with insufficient quantity or DNA yield, and no plasma sample failed technically. These generated no interpretable result and can contribute neither a positive nor a negative call. Second, 16 technically successful pairs in which one or both modalities reported no somatic alteration were excluded, because a paired concordance statistic cannot be computed where one modality reports nothing to compare against. These are informative negative results rather than test failures, and both groups are reinstated in the sensitivity analyses described in Section 2.5. Thus, the analysis estimates the paired-platform detection yield in a selected, matched, evaluable cohort.

Paired testing was defined as tissue and plasma CGP performed during the same clinical testing episode, with tissue biopsy and plasma blood draw occurring within 30 days of each other. The plasma time point was the blood draw date, and archival tissue specimens collected more than 30 days before the plasma blood draw were excluded. An exploratory non-NSCLC subgroup excluded patients with NSCLC and repeated the same patient-level yield analysis.

### 2.2. Comprehensive Genomic Profiling Assays

Plasma CGP was performed using Guardant360, and tissue CGP was performed using Guardant360 TissueNext (Guardant Health, Inc., Redwood City, CA, USA). Plasma CGP interrogated up to 83 genes (v2.12) and tissue CGP up to 498 genes (v1.5); both panel versions were in routine clinical use throughout the study period. Both assays report clinically relevant genomic alteration classes, including single-nucleotide variants, insertions and deletions, fusions, copy-number amplifications, tumor mutational burden (TMB), and microsatellite instability (MSI) status [33,34,35].

The primary paired-platform comparison was restricted to genes common to both assays and shared biomarker outputs; assay-specific content was excluded. Because the plasma panel is contained within the tissue panel, the shared content comprised all 83 plasma genes together with TMB and MSI status. TMB-high was defined as blood TMB ≥ 20 mutations per megabase (mut/Mb) and tissue TMB ≥ 10 mut/Mb. TMB was computed by the manufacturer’s algorithm across a coding footprint of 2.0 Mb for plasma and 2.1 Mb for tissue. Samples not meeting the assay’s tumor-shedding or unique-molecule-coverage criteria were reported as TMB-unevaluable according to the manufacturer’s specification. Germline and somatic variants were distinguished by the manufacturer’s bioinformatic classification; paired leukocyte sequencing was not performed. Because blood and tissue TMB are derived from different analytes, different panels, and use different thresholds, they were treated as modality-specific biomarker outputs rather than directly interchangeable quantitative measurements. Laboratory turnaround time was defined as the interval in calendar days from receipt of the sample in the testing laboratory to issue of the final report. It does not include the pre-analytical interval from biopsy or phlebotomy to sample receipt, nor the interval from report issue to clinical review; therefore, it underestimates the total clinical testing interval experienced by the patient. Turnaround time was calculated to the date of the first final report, and technically unsuccessful reports were excluded from the turnaround-time analysis. Five tissue reports were subsequently amended or corrected (Supplementary Table S5); for these, the final issued version was used for alteration calls, and the date of the first final report was used for turnaround time.

### 2.3. OncoKB Classification and Cancer-Type Harmonization

Reported alterations and biomarker results were annotated by matching gene or biomarker, alteration and cancer type against the OncoKB biomarker–drug associations table [36] (release v6.2, accessed 31 March 2026), using somatic-setting associations only. That table does not distinguish Level 3A from Level 3B, so Level 3 findings are reported without sublevel. Reported diagnoses were harmonized to the closest clinically appropriate OncoKB disease label; nonspecific diagnoses, including cancer of unknown primary, were mapped only to tumor-agnostic categories unless a more specific diagnosis was available.

The primary endpoint was patient-level detection of at least one clinically relevant finding by tissue CGP, plasma CGP, or both, defined as an OncoKB therapeutic Level 1, Level 2 or R1 alteration, or a TMB-high or MSI-high result reported as positive. Annotation was restricted a priori to these levels, with therapeutic Level 3 recorded as an exploratory tier; Level 4, R2, and diagnostic (Dx1–Dx3) and prognostic (Px1–Px3) associations were not recorded. The same annotation scope and cancer-type mapping were applied to tissue and plasma reports. All annotated findings are listed in Supplementary Table S2.

Each patient was counted once per modality and assigned to the overlap, tissue-only or plasma-only group according to which modalities returned a clinically relevant finding. Single-copy copy-number loss was counted only where the report specified biallelic loss, exon-level deletion, or a co-occurring loss-of-function event consistent with the relevant annotation.

### 2.4. Molecularly Informed Therapy Assessment

Molecularly informed therapy was assessed descriptively among patients with available post-CGP treatment regimen data. Therapy was considered molecularly informed if it was aligned with tissue and/or plasma NGS findings, including the use of a genomically matched agent, avoidance of therapy predicted to be ineffective by a resistance biomarker, or molecularly guided standard-of-care treatment. Therapy was classified as not molecularly informed (unmatched) when treatment selection did not align with detected alterations or with the guideline-based interpretation of the molecular report. Among patients receiving molecularly informed therapy, the modality primarily informing the decision was classified using pre-specified criteria. A decision was attributed to a modality where the alteration targeted by the administered therapy was reported by that modality only, or where that modality’s report was the only one available to the treating physician on or before the documented date of the treatment decision. Where both reports were available before the decision and both reported the targeted alteration, the report issued first was recorded as the basis for attribution. Cases with documentation insufficient to establish which report informed the decision were classified as indeterminate and excluded from both the treatment-alignment denominator and the attribution denominator, because the available documentation was insufficient to establish either whether the therapy administered was molecularly informed or which report informed it. Attribution identifies which report was temporally and genomically positioned to inform a decision; it does not establish that the therapy would not otherwise have been given.

Molecularly informed therapy was evaluated only as a treatment-alignment endpoint. Response, progression-free survival, and overall survival data were not available for this analysis. Turnaround time was summarized descriptively.

### 2.5. Exploratory Sensitivity Analyses

Three post hoc sensitivity analyses examined how far the primary result depended on how the analysis cohort and the endpoint were defined.

Two analyses addressed cohort construction. In the first, all technically successful paired tests were analyzed (*n* = 139), reinstating the 16 pairs in which one modality reported no somatic alteration. In the second, all 158 paired sample sets were analyzed, additionally reinstating the 19 patients whose tissue report was technically unsuccessful; for these, tissue was classified as endpoint-negative because no tissue result exists, while the paired plasma report was analyzed normally. Alterations in the reinstated pairs were annotated using the same cancer-type-specific OncoKB criteria applied to the primary cohort. Every one of the 158 paired sample sets is therefore represented in at least one analysis, and no value is imputed. Classifying an unsuccessful tissue test as endpoint-negative in this analysis reflects failure of the tissue-testing strategy to deliver a result, that is, a sample-viability problem, and is not an interpretable biological negative: no tissue result exists to interpret, and no value was imputed for these samples. This analysis therefore estimates the yield of a testing strategy as it would perform in practice, rather than comparing two interpretable results.

The third analysis addressed endpoint composition. The primary endpoint was recalculated in the matched evaluable cohort after excluding TMB-high and MSI-high status, so that only OncoKB Level 1, Level 2 and R1 alterations contributed. TMB-high and MSI-high concordance between modalities is additionally reported separately.

### 2.6. Statistical Analysis

Categorical variables are presented as counts and percentages, and continuous variables as medians with interquartile ranges or ranges as indicated. Wilson 95% confidence intervals were calculated for patient-level detection proportions, sensitivity-analysis proportions, and molecularly informed therapy proportions. No formal hypothesis testing was planned because the study was exploratory and not powered for clinical outcomes. Post hoc paired comparisons of tissue and plasma detection rates used the exact McNemar test and are reported for primary and sensitivity analyses only. Because no independent orthogonal reference standard was available, analytic sensitivity, specificity, positive predictive value, and negative predictive value were not calculated. The analysis instead reports detection yield, unique yield, overlap, and the proportion of combined-positive patients detected by each modality.

Generative artificial intelligence was used to prepare Figure 1, Figure 2, Figure 3 and Figure 4. The authors computed all underlying values from the analytic dataset and verified them against Supplementary Table S5 before drawing the figures. No figure represents primary image data; no micrograph or sequencing output was generated or modified; and the tool was not used to formulate the study design, select the statistical methods, generate or alter the underlying data, or interpret the findings. The authors take full responsibility for all figures.

### 2.7. Data Sources and Ethics

De-identified clinical data were abstracted from medical records, and molecular profiling data were abstracted from CGP reports for paired-platform analysis, OncoKB classification, treatment alignment, and assay attribution. The de-identified per-pair analytic dataset is provided as Supplementary Table S5. The study was conducted in accordance with the Declaration of Helsinki and was reviewed by the Institutional Review Board of Fortis Memorial Research Institute, which waived the requirement for informed consent for retrospective analysis of de-identified clinical and molecular data (FIEC Code No. IEC/2026/OAS/023).

## 3. Results

### 3.1. Patient Flow and Cohort Characteristics

Among 158 patients with paired tissue and plasma CGP results, 123 matched evaluable tissue–plasma pairs were included in the analytic cohort; 35 paired cases were excluded according to analytic criteria (Figure 1).

**Figure 1:**
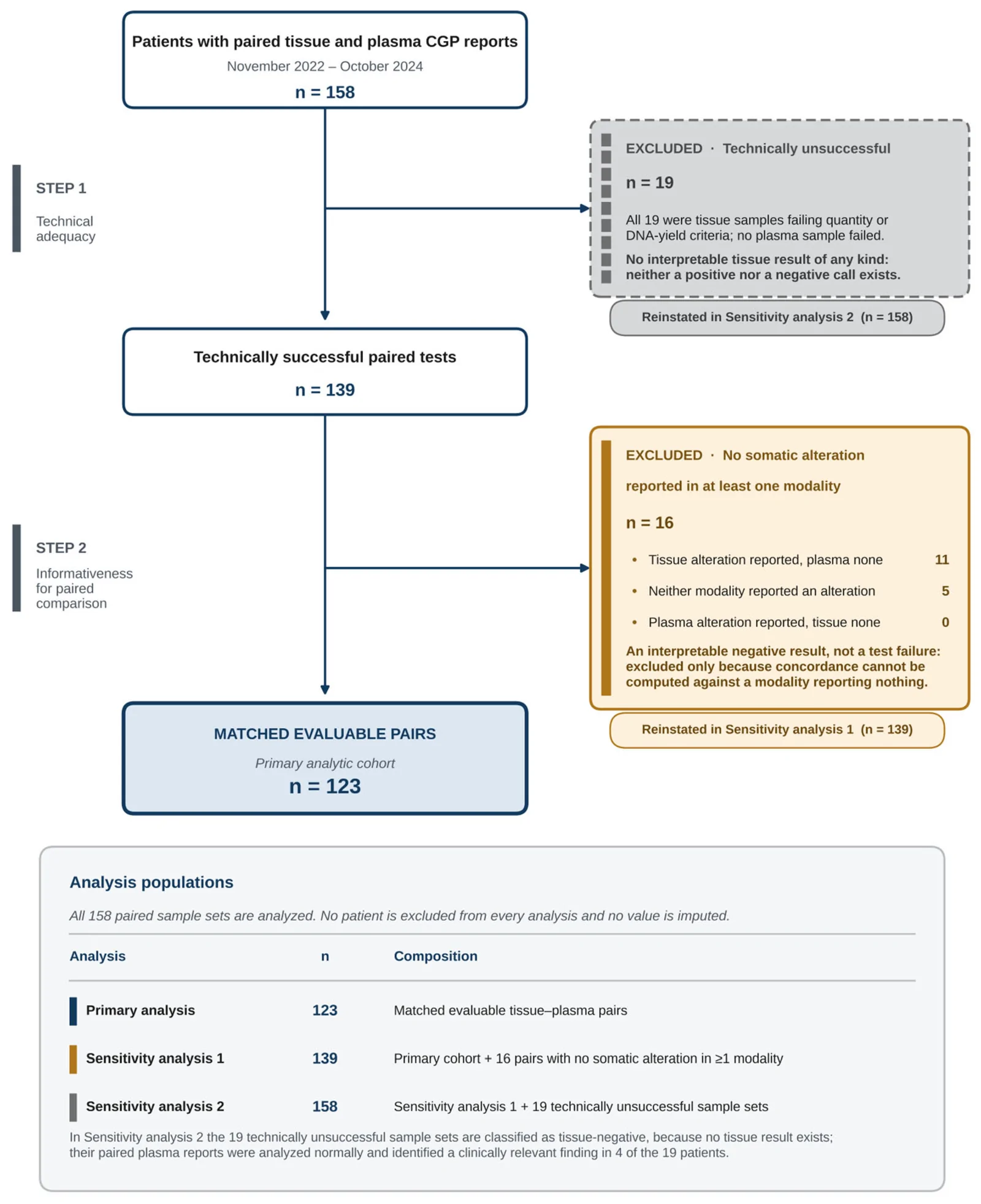
**Study flow diagram.** A total of 158 paired tissue–plasma sample sets were identified, of which 123 matched evaluable pairs formed the primary analytic cohort. The 35 remaining sample sets fall into two categories with different implications for interpretation, which are shown separately. Step 1, technical adequacy: 19 sample sets were technically unsuccessful; all 19 were tissue samples failing quantity or DNA-yield criteria, and no plasma sample failed technically. These generated no interpretable tissue result of any kind, so they can contribute neither a positive nor a negative call. Step 2, informativeness for paired comparison: 16 pairs were technically successful but reported no somatic alteration in at least one modality—11 in which a tissue alteration was reported and no plasma alteration, 5 in which neither modality reported an alteration, and none in which a plasma alteration was reported without a tissue alteration. An absence of reported alterations is an interpretable negative result and not a test failure; these pairs were excluded from the primary cohort only because a paired concordance statistic cannot be computed where one modality reports nothing to compare against. Both groups are reinstated in the exploratory sensitivity analyses (Section 3.4; Supplementary Table S1), so that all 158 paired sample sets are analyzed and no value is imputed. In Sensitivity analysis 2, the 19 technically unsuccessful sample sets are classified as tissue-negative because no tissue result exists, while their paired plasma reports were analyzed normally; plasma identified a clinically relevant finding in 4 of these 19 patients. Dashed outline, no interpretable result exists; solid outline, an interpretable negative result. CGP, comprehensive genomic profiling.

The median age was 62 years (range 4–88). Of the 123 patients, 71 (57.7%) were male, and 52 (42.3%) were female; all had stage IV disease. Line-of-therapy data were available for 118 patients: 50 (42.4%) were tested in the first-line setting, 41 (34.7%) in the second-line setting, 19 (16.1%) in the third-line setting, and 8 (6.8%) after more than three prior lines of therapy (Table 1).

Tumor types included non-small cell lung cancer (NSCLC) in 17 patients (13.8%), other gastrointestinal cancers in 15 (12.2%), pancreatic cancer in 13 (10.6%), breast cancer in 12 (9.8%), colorectal cancer in 11 (8.9%), genitourinary cancers in 10 (8.1%), gynecologic cancers in 9 (7.3%), and other cancers in 36 (29.3%) (Table 1).

### 3.2. Clinically Relevant OncoKB/TMB-High/MSI-High Findings by Tissue and Plasma CGP

Using the prespecified endpoint definition, 46 of 123 patients had at least one clinically relevant finding detected by tissue CGP, plasma CGP, or both modalities (37.4%; 95% CI, 29.4% to 46.2%).

Tissue CGP detected clinically relevant findings in 33 patients (26.8%; 95% CI, 19.8% to 35.3%), and plasma CGP detected clinically relevant findings in 35 patients (28.5%; 95% CI, 21.2% to 37.0%). Twenty-two patients had findings detected by both modalities. The remaining 24 of 46 combined-positive patients (52.2%) had findings detected by only one modality: 11 tissue-only and 13 plasma-only cases (Table 2 and Figure 2).

**Figure 2:**
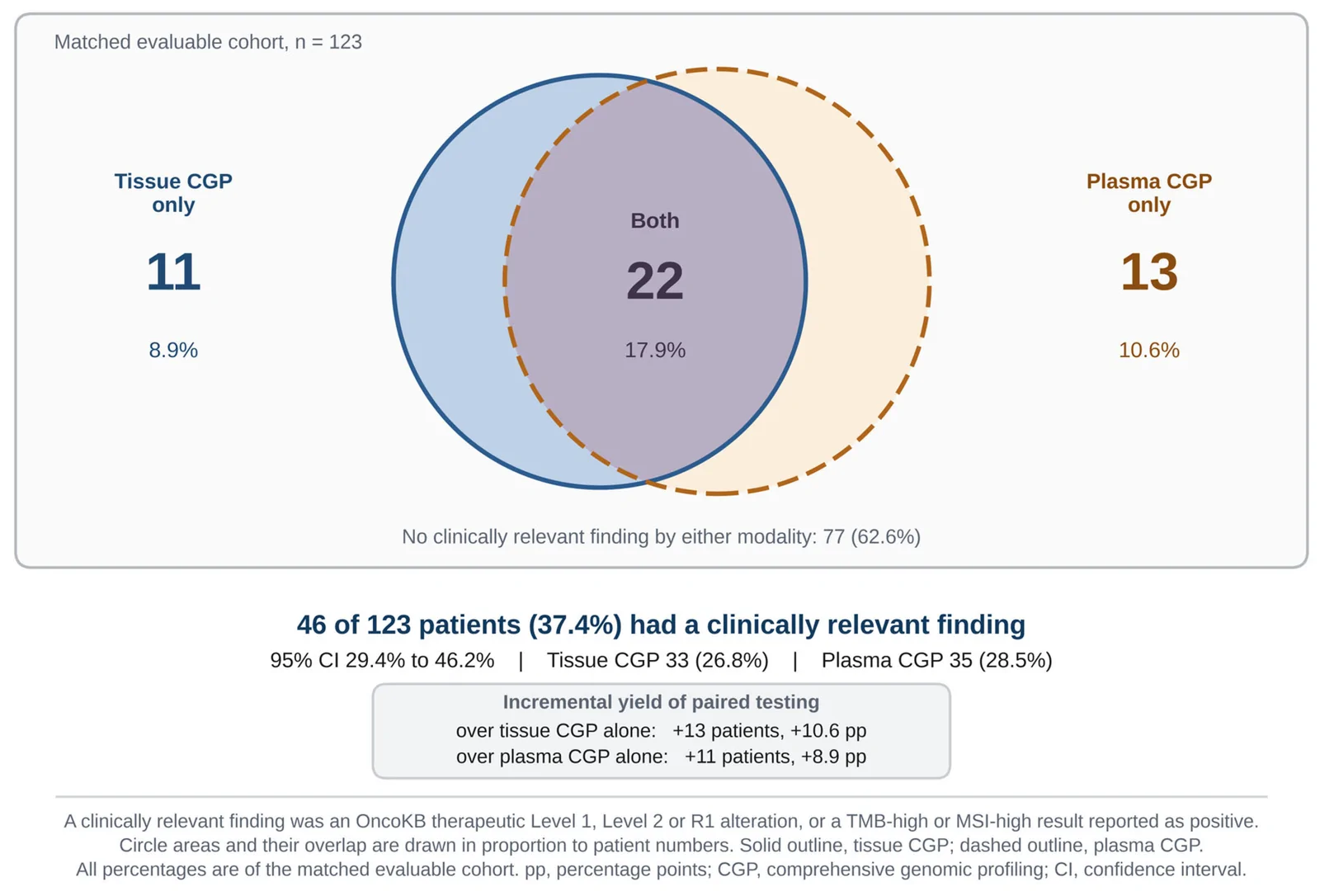
**Patient-level detection of clinically relevant findings by tissue and plasma CGP.** A clinically relevant finding was detected by either modality in 46 of the 123 patients in the matched evaluable cohort (37.4%; 95% CI, 29.4% to 46.2%), by tissue CGP in 33 (26.8%), by plasma CGP in 35 (28.5%) and by both in 22 (17.9%); 11 patients (8.9%) had tissue-only findings, 13 (10.6%) had plasma-only findings, and 77 (62.6%) had no clinically relevant finding by either modality. A clinically relevant finding was an OncoKB therapeutic Level 1, Level 2 or R1 alteration, or a TMB-high or MSI-high result reported as positive. The enclosing frame represents the full matched evaluable cohort, so patients outside both circles are those with no clinically relevant finding. Circle areas and their overlap are drawn in proportion to patient numbers. Solid outline, tissue CGP; dashed outline, plasma CGP. pp, percentage points; CGP, comprehensive genomic profiling; CI, confidence interval; MSI, microsatellite instability; TMB, tumor mutational burden.

Within the combined-positive group, 33 of 46 patients (71.7%) were detected by tissue CGP and 35 of 46 (76.1%) by plasma CGP. Compared with tissue CGP alone, paired testing identified 13 additional patients, corresponding to a 10.6 percentage-point absolute increase and a 39.4% relative increase. Compared with plasma CGP alone, paired testing identified 11 additional patients, corresponding to an 8.9 percentage-point absolute increase and a 31.4% relative increase.

The composite endpoint drew on both genomic alterations and immunotherapy biomarkers. Of the 46 combined-positive patients, 27 (58.7%) qualified through an OncoKB Level 1, Level 2 or R1 alteration and 27 (58.7%) through TMB-high or MSI-high status, with 8 patients qualifying through both. TMB-high or MSI-high status was detected by either modality in 27 patients but by both modalities in only 6 (Supplementary Table S6). Fifteen of the 24 patients with a finding in one modality only (62.5%) were modality-exclusive solely because of a discordant TMB-high call: 9 of 13 plasma-only and 6 of 11 tissue-only patients. Tissue TMB was not evaluable or not reported in 16 of 123 pairs and plasma TMB in 30 of 123, and these were counted as not TMB-high. The endpoint was recalculated without TMB-high and MSI-high as an exploratory sensitivity analysis (Section 3.4).

### 3.3. Exploratory Non-NSCLC Subgroup Analysis

After excluding 17 patients with NSCLC, 106 patients remained in the exploratory non-NSCLC subgroup. Clinically relevant findings were detected by either modality in 35 of 106 patients (33.0%; 95% CI, 24.8% to 42.4%). Tissue CGP detected findings in 24 patients (22.6%), plasma CGP in 27 (25.5%), and both modalities in 16 (15.1%). Eight patients had tissue-only findings, and 11 had plasma-only findings (Supplementary Table S7).

Compared with tissue CGP alone, paired testing identified 11 additional patients, corresponding to a 10.4 percentage-point absolute increase and a 45.8% relative increase. Compared with plasma CGP alone, paired testing identified 8 additional patients, corresponding to a 7.5 percentage-point absolute increase and a 29.6% relative increase (Figure 3; Supplementary Table S7).

**Figure 3:**
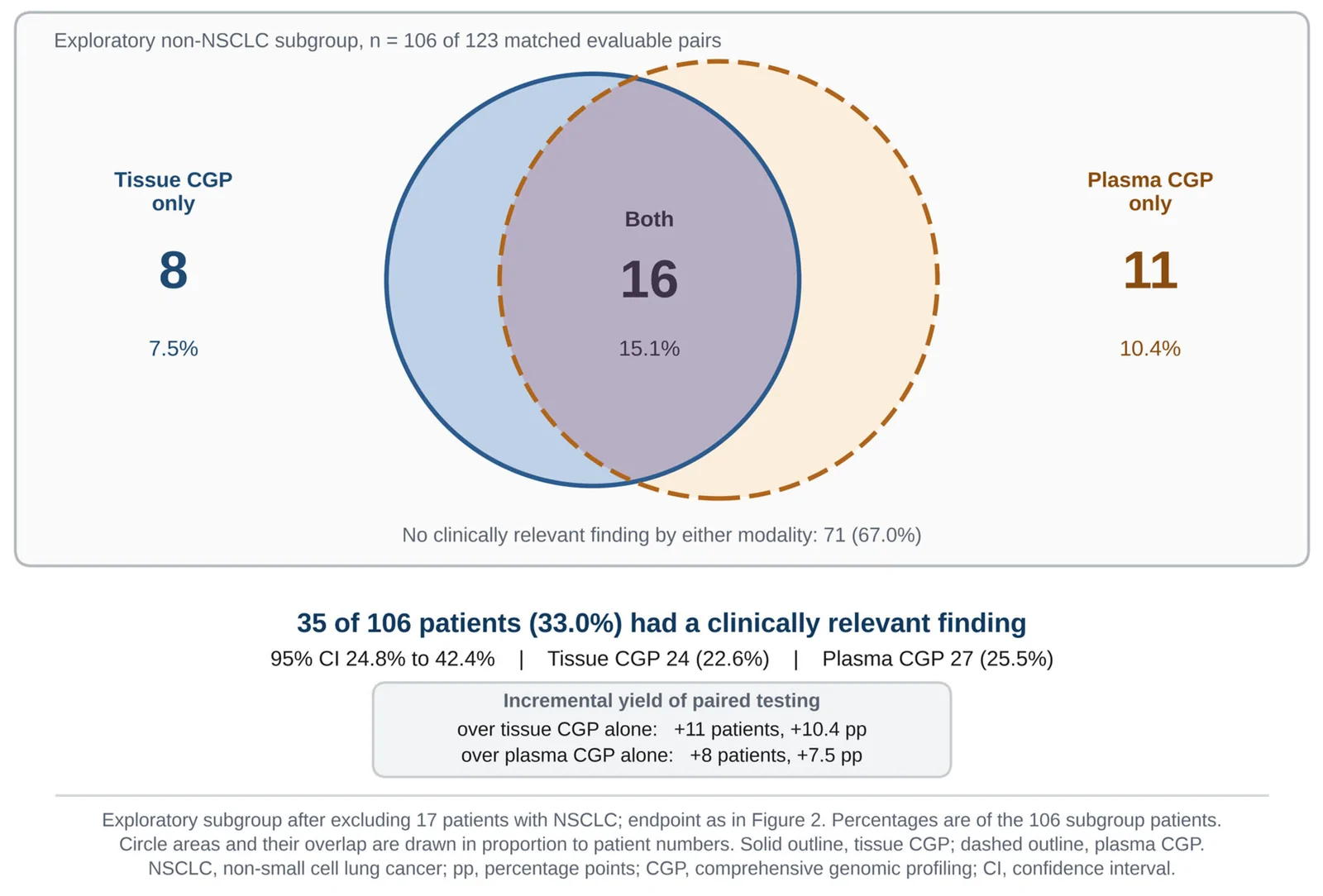
**Patient-level detection of clinically relevant findings by tissue and plasma CGP in the exploratory non-NSCLC subgroup.** After excluding 17 patients with NSCLC, 106 of the 123 matched evaluable pairs remained. A clinically relevant finding, defined as in Figure 2, was detected by either modality in 35 patients (33.0%; 95% CI, 24.8% to 42.4%), by tissue CGP in 24 (22.6%), by plasma CGP in 27 (25.5%) and by both in 16 (15.1%); 8 patients (7.5%) had tissue-only findings, 11 (10.4%) had plasma-only findings, and 71 (67.0%) had no clinically relevant finding by either modality. Paired testing detected findings in 11 more patients than tissue CGP alone (+10.4 percentage points) and 8 more than plasma CGP alone (+7.5 percentage points). This analysis was exploratory and the subgroup was not powered for formal comparison. Conventions as in Figure 2. Corresponding counts are given in Supplementary Table S7. NSCLC, non-small cell lung cancer.

### 3.4. Yield Across Exploratory Sensitivity Analyses

The incremental yield of paired testing persisted when the excluded sample sets were reinstated (Supplementary Table S1). Analyzing all 139 technically successful paired tests, a clinically relevant finding was detected in 34 patients (24.5%) by tissue CGP and 35 (25.2%) by plasma CGP, with 47 patients (33.8%) positive by either modality; 13 patients (9.4%) had plasma-only findings and 12 (8.6%) tissue-only findings. Analyzing all 158 paired sample sets, including the 19 patients whose tissue report was technically unsuccessful, 34 patients (21.5%) were positive by tissue CGP and 39 (24.7%) by plasma CGP, with 51 (32.3%) positive by either modality, 17 (10.8%) plasma-only and 12 (7.6%) tissue-only.

Eight additional clinically relevant alterations in five patients were identified among the previously excluded pairs, all genomic: KIT W557_V559delinsC in a gastrointestinal stromal tumor (tissue), and in plasma ATM biallelic loss with a CUL5-ATM fusion together with AR T878A and AR amplification in prostate adenocarcinoma, KRAS G13D and KRAS G12C in two colorectal adenocarcinomas, and EGFR L747_A750delinsP in a lung adenocarcinoma (Supplementary Table S2). None of the previously excluded pairs was TMB-high or MSI-high in either modality. Four of these five patients had a technically unsuccessful tissue report, so plasma CGP was the only source of genomic information available to the treating physician.

The third sensitivity analysis addressed endpoint composition. Excluding TMB-high and MSI-high status and restricting the endpoint to OncoKB Level 1, Level 2 and R1 alterations, 27 of 123 patients (22.0%) were positive by either modality: 23 (18.7%) by tissue CGP, 21 (17.1%) by plasma CGP, 17 by both modalities, 6 (4.9%) by tissue only and 4 (3.3%) by plasma only (Supplementary Table S1). Compared with tissue CGP alone, paired testing identified 4 additional patients, a 3.3 percentage-point absolute increase; compared with plasma CGP alone, 6 additional patients, a 4.9 percentage-point absolute increase. Bidirectional complementarity was therefore preserved without the immunotherapy biomarkers, although its magnitude was smaller.

Tissue and plasma detection rates did not differ significantly in the primary analysis or in any sensitivity analysis (exact McNemar *p* = 0.84, 1.00, 0.46 and 0.75, respectively), consistent with complementarity between the two modalities rather than superiority of either. A complete listing of every annotated finding, with tumor type, gene, alteration, OncoKB level, and detecting modality, is provided in Supplementary Table S2.

### 3.5. Molecularly Informed Therapy and Turnaround Time

We retrieved treatment records for 97 of 123 patients (78.9%). In six of these, documentation was insufficient to determine either whether the therapy administered was molecularly informed or which report informed the decision; these six patients were therefore classified as indeterminate and excluded from both the treatment-alignment denominator and the attribution denominator. Therapy-alignment data were therefore available and classifiable for 91 of 123 patients (74.0%). Overall, 25 of 91 patients (27.5%; 95% CI, 19.4% to 37.4%) received molecularly informed therapy, and 66 of 91 (72.5%; 95% CI, 62.6% to 80.6%) did not (Table 3; Figure 4).

**Figure 4:**
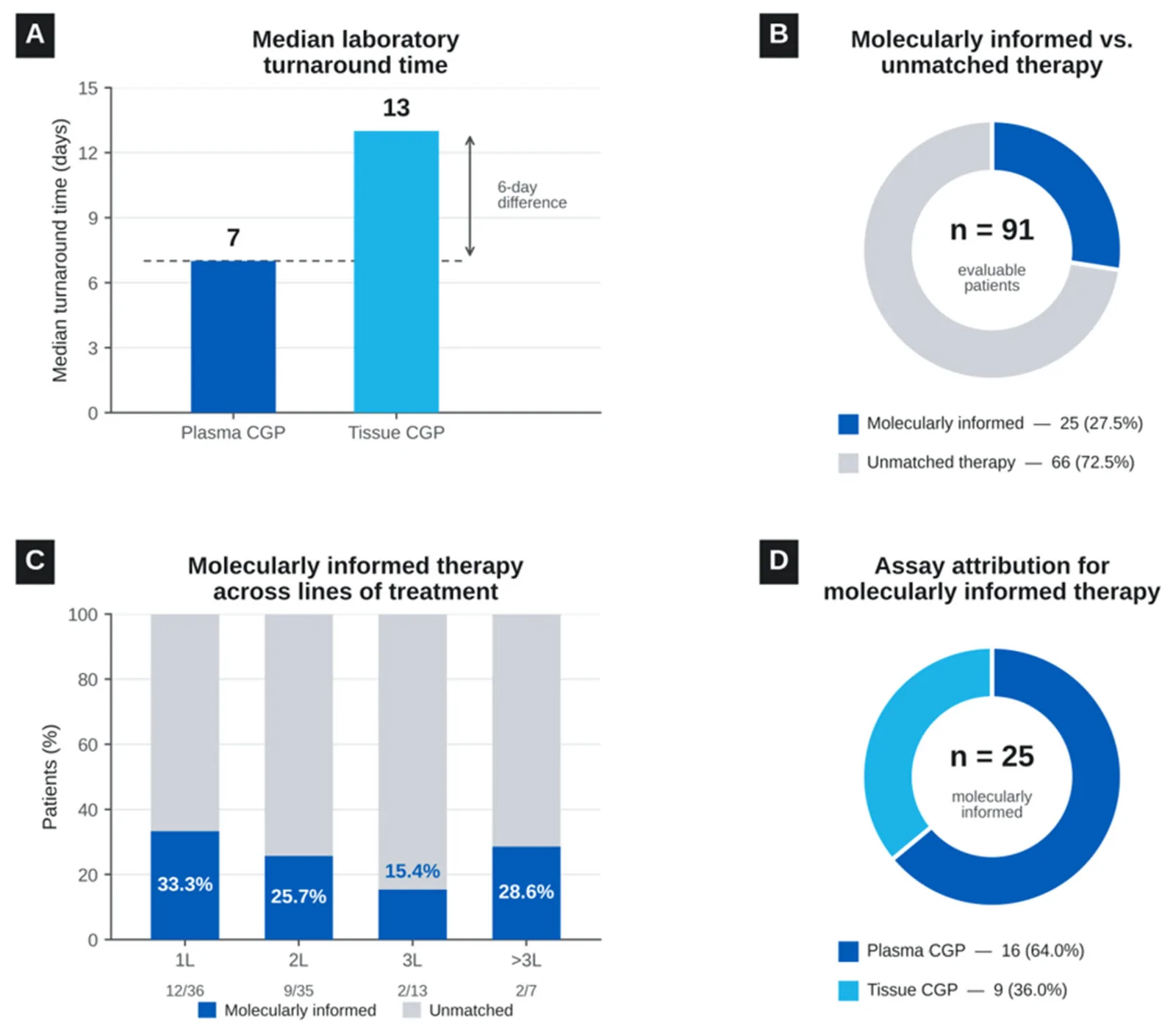
Therapy-alignment data were available and classifiable for 91 of 123 patients (74.0%); an additional 6 patients had treatment records insufficient either to classify therapy alignment or to attribute a decision, and were excluded from both denominators. Molecularly informed versus unmatched therapy and turnaround time after paired tissue and plasma CGP. (**A**) Median turnaround time for plasma and tissue CGP: 7 and 13 days, respectively. (**B**) Molecularly informed versus unmatched therapy among patients with therapy-alignment data: 25 of 91 (27.5%) received molecularly informed therapy, and 66 of 91 (72.5%) received unmatched therapy. (**C**) Molecularly informed therapy by line of treatment: 12 of 36 (33.3%) in first line, 9 of 35 (25.7%) in second line, 2 of 13 (15.4%) in third line, and 2 of 7 (28.6%) beyond third line. (**D**) Assay attribution for molecularly informed therapy was to plasma CGP in 16 of 25 patients (64.0%) and tissue CGP in 9 of 25 patients (36.0%).

By line of therapy, molecularly informed therapy was received by 12 of 36 patients (33.3%) in the first-line setting, 9 of 35 (25.7%) in the second-line setting, 2 of 13 (15.4%) in the third-line setting, and 2 of 7 (28.6%) beyond the third-line setting.

Among the 25 patients receiving molecularly informed therapy, the treatment decision was attributed primarily to plasma CGP findings in 16 patients (64.0%; 95% CI, 44.5% to 79.8%) and to tissue CGP findings in 9 patients (36.0%; 95% CI, 20.2% to 55.5%). Median laboratory turnaround time from sample receipt to report date was 7 days (IQR, 6 to 8; *n* = 115 reports with dates) for plasma CGP and 13 days (IQR, 8 to 16; *n* = 111) for tissue CGP. Among the 110 patients for whom both report dates were available, the plasma report was issued first in 94 (85.5%), on the same day in 6, and after the tissue report in 10 (Supplementary Table S4).

## 4. Discussion

In this retrospective paired-sample study of patients with stage IV solid tumors, tissue and plasma CGP provided complementary, modality-specific detection of clinically relevant genomic findings. Plasma CGP identified additional findings beyond tissue CGP alone, whereas tissue CGP identified findings not detected in plasma. These results support a paired-testing model in which tissue and plasma are best viewed as complementary rather than interchangeable.

The two TMB thresholds applied here differ because blood and tissue TMB are different measurements. Even when derived from the same hybridization-capture panel, the two algorithms count different variant classes at different allele-frequency floors: tissue TMB includes single-nucleotide variants together with insertions and deletions at an allele frequency of 5% or more, whereas blood TMB counts single-nucleotide variants alone down to 0.5% [37]. The two measurements also sample different tumor territory. In 121 patients with matched primary and metastatic tissue sequenced on the same panel, metastatic TMB was 36% higher than primary TMB, and the high-versus-low classification at a 10 mut/Mb threshold was discordant between the two specimens in 9.9% [38]. A tissue score therefore reflects the lesion sampled, whereas plasma integrates shedding from all lesions and can capture heterogeneity that a single specimen misses. Conversely, blood TMB is influenced by non-tumor cell-free DNA, and tissue–plasma concordance falls as tumor fraction falls; when the same cell-free DNA sample is run through both algorithms, agreement is high, indicating that most of the divergence arises from the specimen rather than from the assay [37]. These two specimen-level effects act in opposite directions, so blood TMB should not be assumed to be a systematic under- or over-estimate of tissue TMB in an individual patient. A blood threshold therefore has to be established empirically against outcome in blood rather than transposed from tissue.

The tissue threshold of 10 mut/Mb follows the tumor-agnostic pembrolizumab indication supported by KEYNOTE-158, in which 102 of 790 evaluable patients (13%) were TMB-high and the objective response rate was 29% (95% CI, 21% to 39%) [39]. For blood, three independent datasets converge above that value: 16 mutations across a 1.1 Mb panel, approximately 14.5 mut/Mb, in POPLAR and OAK [37]; 20 mut/Mb in MYSTIC [40]; and 19 mut/Mb in an East Asian cohort profiled on the same platform as MYSTIC [41]. We adopted 20 mut/Mb, the most stringent of these, accepting reduced sensitivity in exchange for a more specific call. Thresholds nonetheless remain platform-specific, and transferability was not tested in this cohort.

The nearest published comparator, the multicancer cohort of Iams et al., differs from ours in almost every methodological respect: 3209 patients across four cancer types, different sequencing assays, and actionability defined by indication-matched NCCN gene-level variants rather than OncoKB levels [29]. Of their patients, 1448 (45.1%) were positive by either assay; within that group, 24.2% had a variant found only in tissue and 9.3% only in plasma. On the same denominator, 11 of our 46 combined-positive patients (23.9%) had tissue-only and 13 (28.3%) plasma-only findings. The tissue-only fractions agree closely. Our plasma-only fraction is higher, but largely for a definitional reason: TMB-high status lies outside their framework, and 9 of our 13 plasma-only patients were plasma-only solely because of a discordant TMB-high call. Restricting our endpoint to OncoKB alterations gives 22.2% (6 of 27) and 14.8% (4 of 27). That two cohorts this dissimilar arrive at similar unique-detection fractions suggests bidirectional complementarity is a property of paired testing rather than of a particular assay pair, tumor mix or health system.

The exploratory non-NSCLC subgroup analysis supports the pan-cancer relevance of these findings. After excluding patients with NSCLC, paired testing continued to increase patient-level detection compared with either modality alone, with both plasma-only and tissue-only findings observed. This suggests that the added yield of paired testing was not driven solely by NSCLC, the disease setting in which concurrent tissue–plasma profiling is most established, and may extend to broader solid-tumor practice.

The exploratory sensitivity analysis did show that incremental yield of paired testing was sensitive to the thresholds adopted—restricting the endpoint to OncoKB Level 1, Level 2 and R1 alterations, 27 of 123 patients (22.0%) remained positive by either modality, with 6 of 123 (4.9%) tissue-only and 4 of 123 (3.3%) plasma-only findings. Complementarity was therefore preserved even without the immunotherapy biomarkers, at a smaller magnitude.

The distribution of MSI-high or TMB-high findings has practical implications. Of the 27 patients with TMB-high or MSI-high status by either modality, 16 had tumor types for which immune checkpoint inhibition is not an established standard-of-care option, including microsatellite-stable colorectal, prostate, pancreatic, duodenal, thyroid, ovarian and neuroendocrine carcinoma; in 12 of these 16 the biomarker was detected by one modality only. For these patients, CGP raised an immunotherapy option that tumor-type-directed treatment algorithms alone would not have surfaced, subject to the line-of-therapy conditions of the tumor-agnostic indication. Concordance for these biomarkers was nonetheless limited: 27 patients were positive by either modality but only 6 by both.

The molecularly informed therapy and turnaround-time analyses connect molecular yield with clinical workflow. Among patients with available treatment-alignment data, 27.5% received molecularly informed therapy. This endpoint was descriptive and included direct genomically matched treatment, avoidance of therapy predicted to be ineffective based on resistance biomarkers, and molecularly guided standard-of-care treatment. Plasma CGP accounted for nearly two-thirds of assay attribution among these decisions and returned reports a median of 6 days earlier than tissue CGP. This inference is limited to treatment selection. Because no response or survival endpoint was analyzed and treatment documentation was available for only three quarters of the cohort, these data cannot establish that paired testing improved treatment decisions or patient outcomes. These observations suggest that plasma CGP may contribute both by identifying unique findings and by providing earlier information for time-sensitive treatment decisions. However, tissue-only findings reinforce that plasma testing should not replace tissue CGP when adequate tissue profiling is feasible.

This study contributes real-world pan-cancer data from India, where published paired tissue–plasma CGP experience remains limited. The cohort included gastrointestinal, pancreatic, breast, colorectal, genitourinary, gynecologic, and rare cancers treated at a tertiary cancer center. The value of this study is not to establish one assay as superior, but to quantify what may be gained or missed when either modality is used alone. This framing is relevant for centers deciding whether molecular testing should proceed sequentially or as a paired strategy when timely genomic information is needed.

Several limitations should be emphasized. First, this was a retrospective single-center study, and patients selected for paired testing may differ from those tested with tissue or plasma alone. Second, the primary analysis was restricted to a selected matched evaluable cohort. Reinstating all technically successful paired tests, and then all 158 paired sample sets with technically unsuccessful tissue reports classified as endpoint-negative (a sample-viability failure rather than an interpretable biological negative), reduced the proportion of patients positive by either modality from 37.4% to 33.8% and 32.3% respectively, while the incremental yield of paired testing persisted in both directions in every analysis (Supplementary Table S1). The primary estimate therefore does not materially overstate the incremental yield relative to a fuller paired-testing population, although it remains derived from a cohort selected to have undergone paired testing. Third, the site of the tissue biopsy, primary or metastatic, was not recorded in the analysis dataset, so its contribution to tissue–plasma TMB discordance could not be assessed; the 30-day pairing window makes an archival primary specimen less likely but does not exclude it. Tumor fraction and ctDNA shedding metrics were not captured in the analysis dataset, so the contribution of low shedding to tissue-only findings could not be tested and remains an inference. Fourth, the paired comparison was restricted to shared assay content and may not capture the full informational value of assay-specific genes or biomarkers. Fifth, many tumor-specific sample sizes were small (Supplementary Table S3). Finally, therapy-alignment data were available for only 91 of 123 patients (74.0%), molecularly informed therapy was broadly defined, and response, progression-free survival, and overall survival were not analyzed. The treatment data therefore describe what was prescribed, not what benefited patients, and no inference about clinical benefit can be drawn from them.

The direction of the potential selection bias was toward plasma; all 16 informative-negative exclusions reported no plasma alteration, of which 5 also reported no tissue alteration. The exclusion criterion therefore removed plasma-negative pairs exclusively. Consistent with this, the plasma-over-tissue margin narrowed when all technically successful pairs were analyzed, and the sensitivity analyses in Section 3.4 were performed to bound the effect.

Our discordance estimates carry an important caveat. No orthogonal confirmatory assay was applied to any modality-exclusive call, so we cannot determine which discordant findings represent genuine biological differences between the tumor and the circulating compartment and which represent analytical false negatives or false positives. Both directions have plausible mechanisms. Tissue-only findings may reflect limited ctDNA shedding or low tumor fraction; consistent with this, the only central nervous system findings at any OncoKB level were tissue-only IDH1 R132H alterations in three patients in whom plasma detected other somatic alterations. Plasma-only findings may reflect tumor heterogeneity not captured by a single specimen, as in one colorectal adenocarcinoma with three distinct RAS resistance variants alongside BRAF V600E in plasma only; alternatively, clonal hematopoiesis is a recognized confounder for DNA damage repair gene calls in plasma [22]. Orthogonal confirmation of discordant calls is a priority for prospective work.

Despite these limitations, the study has several strengths. It uses paired tissue and plasma CGP in a real-world pan-cancer cohort, applies cancer-type matched OncoKB classification, incorporates positive TMB-high and MSI-high biomarker results, and reports molecular yield, treatment-alignment attribution, and turnaround time. Overall, the findings support paired tissue and plasma CGP as a complementary strategy when timely and comprehensive molecular assessment is needed, while underscoring the need for prospective studies that include all technically successful paired tests and link testing strategies to treatment delivery and patient outcomes.

## 5. Conclusions

In this retrospective, single-institution paired-sample study from India, paired tissue and plasma CGP recovered partially nonoverlapping clinically relevant findings in a matched evaluable cohort. This result remained consistent even after reinstating all excluded sample sets and excluding the immunotherapy biomarkers from the endpoint. Plasma CGP returned results earlier and identified plasma-only findings, whereas tissue CGP identified tissue-only findings in a comparable proportion of patients. The exploratory non-NSCLC subgroup analysis suggests that the added yield of paired testing extends beyond the disease setting in which concurrent tissue–plasma profiling is most established. Among the subset of patients with treatment documentation, more decisions were attributable to plasma than to tissue findings, although this attribution partly reflects the earlier availability of plasma reports. These observations should be read as proof of concept rather than as evidence of clinical utility. Because no response, progression-free survival or overall survival data were analyzed, these findings describe genomic detection and treatment selection only and support no inference about clinical benefit. Taken together, these findings are consistent with a paired-testing model in which neither tissue nor plasma CGP alone is expected to capture the full spectrum of clinically relevant genomic information, and they provide a rationale for prospective studies that include all technically successful paired tests, confirm discordant calls, and link testing strategy to treatment delivery and patient outcomes.

## Figures and Tables

**Table 1:** Baseline Clinical Characteristics of the Matched Evaluable Paired-Sample Cohort.

Characteristic	Value
Patients with matched evaluable tissue–plasma CGP	123
Median age, years	62 (4–88)
Female	52 (42.3%)
Male	71 (57.7%)
Stage IV disease	123 (100%)
Line of therapy available	118
First line	50/118 (42.4%)
Second line	41/118 (34.7%)
Third line	19/118 (16.1%)
More than third line	8/118 (6.8%)
Non-Small Cell Lung Cancer (NSCLC)	17 (13.8%)
Other gastrointestinal cancers *	15 (12.2%)
Pancreatic cancer	13 (10.6%)
Colorectal cancer	12 (9.8%)
Breast cancer	11 (8.9%)
Genitourinary cancers	10 (8.1%)
Gynecologic cancers	9 (7.3%)
Other cancers †	36 (29.3%)

* Other gastrointestinal cancers include cholangiocarcinoma, gastric, hepatocellular, gallbladder, duodenal, and esophageal or gastroesophageal junction cancers, and gastrointestinal stromal tumors. † Other cancers include central nervous system tumors, sarcomas, squamous cell carcinomas including oral cavity and tongue primaries, neuroendocrine carcinoma, cancer of unknown primary, thyroid cancer, mesothelioma, and other rare tumors, together with cases for which only a nonspecific diagnosis was recorded. Tumor types for individual patients are listed in Supplementary Table S5; tumor-type counts across all 158 paired sample sets are given in Supplementary Table S3. CGP, comprehensive genomic profiling. Percentages are of the 123 patients in the matched evaluable cohort.

**Table 2:** Patient-Level Detection of Clinically Relevant Findings by Tissue CGP, Plasma CGP and Paired Testing (Matched Evaluable Cohort, *n* = 123).

Measure	Patients, *n* (%)	95% CI
Clinically relevant finding detected by either modality	46/123 (37.4%)	29.4 to 46.2
Detected by tissue CGP	33/123 (26.8%)	19.8 to 35.3
Detected by plasma CGP	35/123 (28.5%)	21.2 to 37.0
Detected by both tissue and plasma CGP	22/123 (17.9%)	12.1 to 25.6
Detected by tissue CGP only	11/123 (8.9%)	5.1 to 15.3
Detected by plasma CGP only	13/123 (10.6%)	6.3 to 17.2
Combined-positive patients detected by tissue CGP	33/46 (71.7%)	57.5 to 82.7
Combined-positive patients detected by plasma CGP	35/46 (76.1%)	62.1 to 86.1
Combined-positive patients detected by tissue CGP only	11/46 (23.9%)	13.9 to 37.9
Combined-positive patients detected by plasma CGP only	13/46 (28.3%)	17.3 to 42.5
Incremental yield of paired testing over tissue CGP alone	+13 patients; +10.6 percentage points; +39.4% relative increase	Not calculated
Incremental yield of paired testing over plasma CGP alone	+11 patients; +8.9 percentage points; +31.4% relative increase	Not calculated

A clinically relevant finding was defined for the primary endpoint as an OncoKB therapeutic Level 1, Level 2, or R1 alteration, or a TMB-high or MSI-high result reported as positive. Proportions with 46 as the denominator refer to the combined-positive group and should not be interpreted as analytic sensitivity against an independent reference standard. Percentage points are the difference between the proportion detected by either modality and by the single modality indicated; relative increase expresses the additional patients as a proportion of those detected by that single modality. Non-NSCLC subgroup results are given in Supplementary Table S7. CGP, comprehensive genomic profiling; CI, confidence interval; MSI, microsatellite instability; NSCLC, non-small cell lung cancer; TMB, tumor mutational burden.

**Table 3:** Treatment Decision Impact and Turnaround Time.

Measure	Value	95% CI
Median plasma CGP turnaround time	7 days (IQR, 6 to 8); *n* = 115	Not applicable
Median tissue CGP turnaround time	13 days (IQR, 8 to 16); *n* = 111	Not applicable
Treatment records retrieved	97/123 (78.9%)	Not applicable
Indeterminate, excluded from both denominators	6/97 (6.2%)	Not applicable
Molecularly informed therapy data available	91/123 (74.0%)	Not applicable
Received molecularly informed therapy, overall	25/91 (27.5%)	19.4% to 37.4%
Received unmatched therapy, overall	66/91 (72.5%)	62.6% to 80.6%
Molecularly informed therapy, first line	12/36 (33.3%)	20.2% to 49.7%
Unmatched therapy, first line	24/36 (66.7%)	50.3% to 79.8%
Molecularly informed therapy, second line	9/35 (25.7%)	14.2% to 42.1%
Unmatched therapy, second line	26/35 (74.3%)	57.9% to 85.8%
Molecularly informed therapy, third line	2/13 (15.4%)	4.3% to 42.2%
Unmatched therapy, third line	11/13 (84.6%)	57.8% to 95.7%
Molecularly informed therapy, >third line	2/7 (28.6%)	8.2% to 64.1%
Unmatched therapy, >third line	5/7 (71.4%)	35.9% to 91.8%
Molecularly informed therapy attribution to plasma CGP	16/25 (64.0%)	44.5% to 79.8%
Molecularly informed therapy attribution to tissue CGP	9/25 (36.0%)	20.2% to 55.5%

Molecularly informed therapy included positive or negative matching, or molecularly guided standard-of-care therapy based on tissue and/or plasma NGS. Unmatched therapy was defined as treatment that was not aligned with genomic findings or with guideline-based report interpretation. CI, confidence interval; CGP, comprehensive genomic profiling.

## Data Availability

Restrictions apply to the availability of the data used in this study. De-identified molecular profiling data were obtained from Guardant Health AMEA, and de-identified clinical, demographic, treatment-alignment, and assay-attribution data were obtained from Fortis Memorial Research Institute. Data are not publicly available because of applicable privacy, institutional, and data-use restrictions. The de-identified per-pair analytic dataset supporting all reported analyses is provided as Supplementary Table S5; the underlying source reports and medical records are subject to the restrictions described above. Requests for access may be directed to the corresponding author and will be considered with appropriate approvals, where applicable.

## Supplementary Materials

The following supporting information can be downloaded at: https://www.mdpi.com/article/10.3390/curroncol33100575/s1, Table S1: Exploratory sensitivity analyses of the incremental yield of paired tissue and plasma CGP; Table S2: Clinically relevant alterations detected by tissue CGP, plasma CGP or both; Table S3: Clinically relevant findings by tumor type (all 158 paired sample sets); Table S4: Laboratory turnaround time (sample receipt to issue of final report); Table S5: Per-pair analytic dataset (de-identified, all 158 paired sample sets); Table S6: TMB-high and MSI-high concordance between modalities (*n* = 123); Table S7: Patient-level detection of clinically relevant findings by tissue CGP, plasma CGP and paired testing in the exploratory non-NSCLC subgroup (*n* = 106).

## Abbreviations

The following abbreviations are used in this manuscript:

AMEA	Asia, Middle East, and Africa
CGP	comprehensive genomic profiling
CI	confidence interval
ctDNA	circulating tumor DNA
DNA	deoxyribonucleic acid
Mb	megabase
MSI	microsatellite instability
NGS	next-generation sequencing
NSCLC	non-small cell lung cancer
OncoKB	precision oncology knowledge base
STROBE	Strengthening the Reporting of Observational Studies in Epidemiology
TAT	turnaround time
TMB	tumor mutational burden
