# Supplementary material for: Paired Tissue and Plasma Comprehensive Genomic Profiling in Advanced Solid Tumors: A Retrospective Study from India
Source: Curr Oncol. 2026 Sep 25;33(10):575. doi: 10.3390/curroncol33100575 (PMC13703395; doi:10.3390/curroncol33100575)
Supplement: Supplementary file 1 [file curroncol-33-00575-s001.zip › curroncol-4486051-supplementary.pdf]

Table S1. Exploratory sensitivity analyses of the incremental yield of paired tissue and plasma CGP

Analysis	n	Tissue (n,%; 95% CI)	Plasma (n,%; 95% CI)	Both	Either (n,%; 95% CI)	Tissue only (n,%; 95% CI)	Plasma only (n,%; 95% CI)	McNemar p
Primary analysis	123	33 (26.8%; 19.8–35.3)	35 (28.5%; 21.2–37.0)	22	46 (37.4%; 29.4–46.2)	11 (8.9%; 5.1–15.3)	13 (10.6%; 6.3–17.2)	0.84
E1 — all technically successful paired tests	139	34 (24.5%; 18.1–32.2)	35 (25.2%; 18.7–33.0)	22	47 (33.8%; 26.5–42.0)	12 (8.6%; 5.0–14.5)	13 (9.4%; 5.5–15.3)	1
E2 — all paired sample sets	158	34 (21.5%; 15.8–28.6)	39 (24.7%; 18.6–32.0)	22	51 (32.3%; 25.5–39.9)	12 (7.6%; 4.4–12.8)	17 (10.8%; 6.8–16.6)	0.46
E3 — endpoint excluding TMB-high and MSI-high	123	23 (18.7%; 12.8–26.5)	21 (17.1%; 11.4–24.7)	17	27 (22.0%; 15.5–30.1)	6 (4.9%; 2.3–10.2)	4 (3.3%; 1.3–8.1)	0.75

All analyses use the full 158-patient paired cohort as the sampling frame. No value is imputed: every cohort shown is a real, fully analyzed set of patients, and no pair is counted negative without a report supporting it.

The 19 technically unsuccessful tissue reports are counted as tissue-negative in analysis E2 because no tissue result exists for them; their paired plasma reports were analyzed normally.

None of the 35 previously excluded pairs was TMB-high or MSI-high in either modality, so every reinstated finding is a genomic alteration.

The endpoint definition and all biomarker thresholds are unchanged from the original submission. Tissue and plasma detection rates did not differ significantly in any analysis.

The E3 analysis restricts the endpoint to OncoKB Level 1, Level 2 and R1 alterations. Of the 46 patients positive on the full composite endpoint, 27 qualified through a genomic alteration and 27 through TMB-high or MSI-high status, with 8 qualifying through both. Tissue TMB was not evaluable or not reported in 16 of 123 pairs and plasma TMB in 30 of 123; these were counted as not TMB-high.

Exact McNemar test, two-sided.

Table S2. Clinically relevant alterations detected by tissue CGP, plasma CGP or both

Patient	Tumor type	Gene / biomarker	Alteration	OncoKB level	Detected in	Endpoint tier	Cohort
P4	Breast Carcinoma	PIK3CA	R93Q	1	Tissue only	Primary endpoint (L1/2/R1)	Matched evaluable (n=123)
P24	Gallbladder carcinoma	ERBB2	Amplification	2	Tissue only	Primary endpoint (L1/2/R1)	Matched evaluable (n=123)
P30	Breast Carcinoma	ERBB2	S310F	2	Tissue only	Primary endpoint (L1/2/R1)	Matched evaluable (n=123)
P30	Breast Carcinoma	PIK3CA	H1047R	1	Tissue only	Primary endpoint (L1/2/R1)	Matched evaluable (n=123)
P40	Lung adenocarcinoma	EGFR	L858R	1	Tissue only	Primary endpoint (L1/2/R1)	Matched evaluable (n=123)
P45	Lung squamous cell carcinoma	TMB	TMB-high	1	Tissue only	Primary endpoint (L1/2/R1)	Matched evaluable (n=123)
P52	Lung adenocarcinoma	EGFR	R776C	1	Tissue only	Primary endpoint (L1/2/R1)	Matched evaluable (n=123)
P59	Duodenal Adenocarcinoma	TMB	TMB-high	1	Tissue only	Primary endpoint (L1/2/R1)	Matched evaluable (n=123)
P64	Bladder urothelial (transitional cell) carcinoma	FGFR3	Y373C	1	Tissue only	Primary endpoint (L1/2/R1)	Matched evaluable (n=123)
P71	Thyroid carcinoma	TMB	TMB-high	1	Tissue only	Primary endpoint (L1/2/R1)	Matched evaluable (n=123)
P77	Lung adenocarcinoma	EGFR	R776H	1	Tissue only	Primary endpoint (L1/2/R1)	Matched evaluable (n=123)
P86	Urothelial carcinoma	FGFR3	K650E	2	Tissue only	Primary endpoint (L1/2/R1)	Matched evaluable (n=123)
P94	Colorectal adenocarcinoma	TMB	TMB-high	1	Tissue only	Primary endpoint (L1/2/R1)	Matched evaluable (n=123)
P103	Colorectal adenocarcinoma	KRAS	G12D	R1	Tissue only	Primary endpoint (L1/2/R1)	Matched evaluable (n=123)
P114	Lung adenocarcinoma	TMB	TMB-high	1	Tissue only	Primary endpoint (L1/2/R1)	Matched evaluable (n=123)
P120	Esophageal squamous cell carcinoma	TMB	TMB-high	1	Tissue only	Primary endpoint (L1/2/R1)	Matched evaluable (n=123)
P122	Esophageal/Gastroesophageal junction Adenocarcinoma	TMB	TMB-high	1	Tissue only	Primary endpoint (L1/2/R1)	Matched evaluable (n=123)
P141	GIST (Gastrointestinal stromal tumor)	KIT	W557_V559delinsC	1	Tissue only	Primary endpoint (L1/2/R1)	Technically successful (n=139)
P7	Ovarian carcinoma	CCNE1	Amplification	3	Tissue only	Exploratory (L3)	Matched evaluable (n=123)
P54	Lung adenocarcinoma	KRAS	G12A	3	Tissue only	Exploratory (L3)	Matched evaluable (n=123)
P55	Follicular thyroid carcinoma	NRAS	Q61K	3	Tissue only	Exploratory (L3)	Matched evaluable (n=123)
P56	Pancreatic ductal adenocarcinoma	KRAS	G12V	3	Tissue only	Exploratory (L3)	Matched evaluable (n=123)
P58	Pancreatic ductal adenocarcinoma	KRAS	G12D	3	Tissue only	Exploratory (L3)	Matched evaluable (n=123)
P88	Gemistocytic astrocytoma	IDH1	R132H	3	Tissue only	Exploratory (L3)	Matched evaluable (n=123)

Patient	Tumor type	Gene / biomarker	Alteration	OncoKB level	Detected in	Endpoint tier	Cohort
P106	Glioblastoma	IDH1	R132H	3	Tissue only	Exploratory (L3)	Matched evaluable (n=123)
P111	Diffuse midline glioma	IDH1	R132H	3	Tissue only	Exploratory (L3)	Matched evaluable (n=123)
P114	Lung adenocarcinoma	ERBB2	Amplification	3	Tissue only	Exploratory (L3)	Matched evaluable (n=123)
P120	Esophageal squamous cell carcinoma	EGFR	Amplification	3	Tissue only	Exploratory (L3)	Matched evaluable (n=123)
P2	Prostate adenocarcinoma	BRCA2	T2125fs	1	Plasma only	Primary endpoint (L1/2/R1)	Matched evaluable (n=123)
P2	Prostate adenocarcinoma	BRCA2	K2128fs	1	Plasma only	Primary endpoint (L1/2/R1)	Matched evaluable (n=123)
P2	Prostate adenocarcinoma	BRCA2	Deletion, exon 11	1	Plasma only	Primary endpoint (L1/2/R1)	Matched evaluable (n=123)
P2	Prostate adenocarcinoma	BRCA2	Biallelic Loss (Single Copy Deletion + Co-Occurring LOF Variant)	1	Plasma only	Primary endpoint (L1/2/R1)	Matched evaluable (n=123)
P2	Prostate adenocarcinoma	TMB	TMB-high	1	Plasma only	Primary endpoint (L1/2/R1)	Matched evaluable (n=123)
P11	Breast Carcinoma	TMB	TMB-high	1	Plasma only	Primary endpoint (L1/2/R1)	Matched evaluable (n=123)
P14	Colorectal adenocarcinoma	TMB	TMB-high	1	Plasma only	Primary endpoint (L1/2/R1)	Matched evaluable (n=123)
P23	Prostate adenocarcinoma	TMB	TMB-high	1	Plasma only	Primary endpoint (L1/2/R1)	Matched evaluable (n=123)
P33	Prostate adenocarcinoma	TMB	TMB-high	1	Plasma only	Primary endpoint (L1/2/R1)	Matched evaluable (n=123)
P48	Cholangiocarcinoma	TMB	TMB-high	1	Plasma only	Primary endpoint (L1/2/R1)	Matched evaluable (n=123)
P50	Colorectal adenocarcinoma	BRAF	V600E	1	Plasma only	Primary endpoint (L1/2/R1)	Matched evaluable (n=123)
P50	Colorectal adenocarcinoma	KRAS	G12A	R1	Plasma only	Primary endpoint (L1/2/R1)	Matched evaluable (n=123)
P50	Colorectal adenocarcinoma	KRAS	Q61H	R1	Plasma only	Primary endpoint (L1/2/R1)	Matched evaluable (n=123)
P50	Colorectal adenocarcinoma	NRAS	Q61K	R1	Plasma only	Primary endpoint (L1/2/R1)	Matched evaluable (n=123)
P55	Follicular thyroid carcinoma	TMB	TMB-high	1	Plasma only	Primary endpoint (L1/2/R1)	Matched evaluable (n=123)
P57	Neuroendocrine carcinoma	BRCA2	H1003fs	2	Plasma only	Primary endpoint (L1/2/R1)	Matched evaluable (n=123)
P64	Bladder urothelial (transitional cell) carcinoma	TMB	TMB-high	1	Plasma only	Primary endpoint (L1/2/R1)	Matched evaluable (n=123)
P84	Colorectal adenocarcinoma	TMB	TMB-high	1	Plasma only	Primary endpoint (L1/2/R1)	Matched evaluable (n=123)
P89	Squamous cell carcinoma of the tongue	TMB	TMB-high	1	Plasma only	Primary endpoint (L1/2/R1)	Matched evaluable (n=123)
P93	Lung adenocarcinoma	BRAF	V600E	1	Plasma only	Primary endpoint (L1/2/R1)	Matched evaluable (n=123)
P96	Non-small cell lung carcinoma, NOS	TMB	TMB-high	1	Plasma only	Primary endpoint (L1/2/R1)	Matched evaluable (n=123)
P97	Colorectal adenocarcinoma	TMB	TMB-high	1	Plasma only	Primary endpoint (L1/2/R1)	Matched evaluable (n=123)
P108	Ovarian carcinoma	TMB	TMB-high	1	Plasma only	Primary endpoint (L1/2/R1)	Matched evaluable (n=123)
P109	Colorectal adenocarcinoma	TMB	TMB-high	1	Plasma only	Primary endpoint (L1/2/R1)	Matched evaluable (n=123)
P121	Colorectal adenocarcinoma	TMB	TMB-high	1	Plasma only	Primary endpoint (L1/2/R1)	Matched evaluable (n=123)
P138	Prostate adenocarcinoma	AR	T878A	R1	Plasma only	Primary endpoint (L1/2/R1)	All paired sets (n=158)
P138	Prostate adenocarcinoma	AR	Amplification	R1	Plasma only	Primary endpoint (L1/2/R1)	All paired sets (n=158)
P138	Prostate adenocarcinoma	ATM	Biallelic Loss (Homozygous Deletion)	1	Plasma only	Primary endpoint (L1/2/R1)	All paired sets (n=158)
P138	Prostate adenocarcinoma	CUL5-ATM	Fusion	1	Plasma only	Primary endpoint (L1/2/R1)	All paired sets (n=158)
P143	Colorectal adenocarcinoma	KRAS	G13D	R1	Plasma only	Primary endpoint (L1/2/R1)	All paired sets (n=158)
P147	Lung adenocarcinoma	EGFR	L747_A750delinsP	1	Plasma only	Primary endpoint (L1/2/R1)	All paired sets (n=158)
P157	Colorectal adenocarcinoma	KRAS	G12C	1/R1	Plasma only	Primary endpoint (L1/2/R1)	All paired sets (n=158)
P40	Lung adenocarcinoma	TP53	Y220C	3	Plasma only	Exploratory (L3)	Matched evaluable (n=123)

Patient	Tumor type	Gene / biomarker	Alteration	OncoKB level	Detected in	Endpoint tier	Cohort
P49	Carcinoma of unknown primary (CUP)	TP53	Y220C	3	Plasma only	Exploratory (L3)	Matched evaluable (n=123)
P137	Endometrial carcinoma	ERBB2	Amplification	3	Plasma only	Exploratory (L3)	All paired sets (n=158)
P4	Breast Carcinoma	PIK3CA	E545K	1	Both	Primary endpoint (L1/2/R1)	Matched evaluable (n=123)
P9	Cholangiocarcinoma	ERBB2	Amplification	2	Both	Primary endpoint (L1/2/R1)	Matched evaluable (n=123)
P10	Breast Carcinoma	ERBB2	Amplification	1	Both	Primary endpoint (L1/2/R1)	Matched evaluable (n=123)
P11	Breast Carcinoma	PIK3CA	E542K	1	Both	Primary endpoint (L1/2/R1)	Matched evaluable (n=123)
P17	Lung adenocarcinoma	EGFR	E746_T751delinsIP	1	Both	Primary endpoint (L1/2/R1)	Matched evaluable (n=123)
P26	Neuroendocrine carcinoma	TMB	TMB-high	1	Both	Primary endpoint (L1/2/R1)	Matched evaluable (n=123)
P28	Pancreatic ductal adenocarcinoma	BRCA1	E1011*	2	Both	Primary endpoint (L1/2/R1)	Matched evaluable (n=123)
P28	Pancreatic ductal adenocarcinoma	TMB	TMB-high	1	Both	Primary endpoint (L1/2/R1)	Matched evaluable (n=123)
P38	Lung adenocarcinoma	EGFR	S768_D770dup	1	Both	Primary endpoint (L1/2/R1)	Matched evaluable (n=123)
P51	Other	TMB	TMB-high	1	Both	Primary endpoint (L1/2/R1)	Matched evaluable (n=123)
P52	Lung adenocarcinoma	EGFR	L858R	1	Both	Primary endpoint (L1/2/R1)	Matched evaluable (n=123)
P63	Lung adenocarcinoma	TMB	TMB-high	1	Both	Primary endpoint (L1/2/R1)	Matched evaluable (n=123)
P71	Thyroid carcinoma	ARHGEF11-NTRK1	Fusion	1	Both	Primary endpoint (L1/2/R1)	Matched evaluable (n=123)
P71	Thyroid carcinoma	MSI	MSI-high	1	Both	Primary endpoint (L1/2/R1)	Matched evaluable (n=123)
P77	Lung adenocarcinoma	EGFR	L858R	1	Both	Primary endpoint (L1/2/R1)	Matched evaluable (n=123)
P82	Renal Cell Carcinoma	BRAF	V600E	1	Both	Primary endpoint (L1/2/R1)	Matched evaluable (n=123)
P84	Colorectal adenocarcinoma	KRAS	G12C	1	Both	Primary endpoint (L1/2/R1)	Matched evaluable (n=123)
P92	Pancreatic ductal adenocarcinoma	BRCA1	S1253fs	2	Both	Primary endpoint (L1/2/R1)	Matched evaluable (n=123)
P102	Hepatocellular carcinoma	MSI	MSI-high	1	Both	Primary endpoint (L1/2/R1)	Matched evaluable (n=123)
P102	Hepatocellular carcinoma	TMB	TMB-high	1	Both	Primary endpoint (L1/2/R1)	Matched evaluable (n=123)
P107	Lung adenocarcinoma	EGFR	L858R	1	Both	Primary endpoint (L1/2/R1)	Matched evaluable (n=123)
P109	Colorectal adenocarcinoma	BRAF	V600E	1	Both	Primary endpoint (L1/2/R1)	Matched evaluable (n=123)
P112	GIST (Gastrointestinal stromal tumor)	KIT	K558delinsNP	1	Both	Primary endpoint (L1/2/R1)	Matched evaluable (n=123)
P121	Colorectal adenocarcinoma	KRAS	G12D	R1	Both	Primary endpoint (L1/2/R1)	Matched evaluable (n=123)
P8	Endometrial carcinoma	CCNE1	Amplification	3	Both	Exploratory (L3)	Matched evaluable (n=123)
P10	Breast Carcinoma	BRCA2	Y1739*	3	Both	Exploratory (L3)	Matched evaluable (n=123)
P12	Lung adenocarcinoma	KRAS	G12V	3	Both	Exploratory (L3)	Matched evaluable (n=123)
P27	Pancreatic ductal adenocarcinoma	KRAS	G12R	3	Both	Exploratory (L3)	Matched evaluable (n=123)
P28	Pancreatic ductal adenocarcinoma	ERBB2	Amplification	3	Both	Exploratory (L3)	Matched evaluable (n=123)
P63	Lung adenocarcinoma	KRAS	G12S	3	Both	Exploratory (L3)	Matched evaluable (n=123)
P72	Endometrial carcinoma	CCNE1	Amplification	3	Both	Exploratory (L3)	Matched evaluable (n=123)
P92	Pancreatic ductal adenocarcinoma	KRAS	G12R	3	Both	Exploratory (L3)	Matched evaluable (n=123)
P99	Pancreatic adenocarcinoma	KRAS	G12D	3	Both	Exploratory (L3)	Matched evaluable (n=123)
P116	Pancreatic ductal adenocarcinoma	KRAS	G12R	3	Both	Exploratory (L3)	Matched evaluable (n=123)
P119	Ovarian carcinoma	TP53	Y220C	3	Both	Exploratory (L3)	Matched evaluable (n=123)

One row per unique patient-gene-alteration event among findings annotated OncoKB Level 1, 2, 3 or R1 on cancer-type-specific annotation, together with TMB-high and MSI-high biomarker calls.

“1/R1” denotes an alteration carrying both a sensitivity and a resistance annotation in the same tumor type, for example KRAS G12C in colorectal adenocarcinoma.

Table S3. Clinically relevant findings by tumor type (all 158 paired sample sets)

Tumor type	Patients (n)	Tissue	Plasma	Either	%	Plasma only	Tissue only	Technically unsuccessful tissue
Lung adenocarcinoma	17	8	8	10	58.8%	2	2	1
Colorectal adenocarcinoma	14	5	8	10	71.4%	5	2	2
Pancreatic ductal adenocarcinoma	14	2	2	2	14.3%	0	0	4
Breast Carcinoma	11	4	3	4	36.4%	0	1	0
Others	8	1	1	1	12.5%	0	0	0
Endometrial carcinoma	6	0	0	0	0.0%	0	0	1
Glioblastoma	6	0	0	0	0.0%	0	0	0
Prostate adenocarcinoma	6	0	4	4	66.7%	4	0	3
Hepatocellular carcinoma	5	1	1	1	20.0%	0	0	1
Neuroendocrine carcinoma	5	1	2	2	40.0%	1	0	0
Ovarian carcinoma	5	0	1	1	20.0%	1	0	1
Cholangiocarcinoma	4	1	2	2	50.0%	1	0	0
Carcinoma of unknown primary (CUP)	3	0	0	0	0.0%	0	0	1
Ewing sarcoma	3	0	0	0	0.0%	0	0	0
Renal cell carcinoma	3	1	1	1	33.3%	0	0	0
Sarcoma	3	0	0	0	0.0%	0	0	1
Duodenal Adenocarcinoma	2	1	0	1	50.0%	0	1	0
Esophageal squamous cell carcinoma	2	1	0	1	50.0%	0	1	0
Esophageal/Gastroesophageal junction Adenocarcinoma	2	1	0	1	50.0%	0	1	1
GIST (Gastrointestinal stromal tumor)	2	2	1	2	100.0%	0	1	0
Gastric adenocarcinoma	2	0	0	0	0.0%	0	0	0
Meningioma	2	0	0	0	0.0%	0	0	0
Squamous cell carcinoma	2	0	0	0	0.0%	0	0	0
Squamous cell carcinoma of the tongue	2	0	1	1	50.0%	1	0	1
Urothelial carcinoma	2	1	0	1	50.0%	0	1	0
Adenoid cystic carcinoma	1	0	0	0	0.0%	0	0	0
Bladder urothelial (transitional cell) carcinoma	1	1	1	1	100.0%	0	0	0
Cancer, Other	1	0	0	0	0.0%	0	0	0
Cervical squamous cell carcinoma	1	0	0	0	0.0%	0	0	0
Cervical adenocarcinoma	1	0	0	0	0.0%	0	0	0
Diffuse midline glioma	1	0	0	0	0.0%	0	0	0
Follicular thyroid carcinoma	1	0	1	1	100.0%	1	0	0
Gallbladder carcinoma	1	1	0	1	100.0%	0	1	0
Gastroesophageal junction carcinoma	1	0	0	0	0.0%	0	0	0
Gemistocytic astrocytoma	1	0	0	0	0.0%	0	0	0

Tumor type	Patients (n)	Tissue	Plasma	Either	%	Plasma only	Tissue only	Technically unsuccessful tissue
Gliosarcoma	1	0	0	0	0.0%	0	0	0
Langerhans cell histiocytosis (LCH)	1	0	0	0	0.0%	0	0	1
Liposarcoma	1	0	0	0	0.0%	0	0	0
Lung squamous cell carcinoma	1	1	0	1	100.0%	0	1	0
Mesothelioma	1	0	0	0	0.0%	0	0	0
Non-small cell lung carcinoma, NOS	1	0	1	1	100.0%	1	0	0
Oral squamous cell carcinoma	1	0	0	0	0.0%	0	0	0
Pancreatic adenocarcinoma	1	0	0	0	0.0%	0	0	0
Pancreatic carcinoid tumor	1	0	0	0	0.0%	0	0	0
Pancreatic neuroendocrine tumor	1	0	0	0	0.0%	0	0	0
Pleomorphic sarcoma	1	0	0	0	0.0%	0	0	0
Sertoli-Leydig cell tumor	1	0	0	0	0.0%	0	0	0
Sinonasal adenocarcinoma	1	0	0	0	0.0%	0	0	0
Spindle cell carcinoma	1	0	0	0	0.0%	0	0	0
Synovial sarcoma	1	0	0	0	0.0%	0	0	0
Thymic carcinoma	1	0	0	0	0.0%	0	0	1
Thyroid carcinoma	1	1	1	1	100.0%	0	0	0
Total	158	34	39	51	32.3%	17	12	19

52 distinct tumor types were represented across the 158 paired sample sets; the median number of patients per type was 1 (range 1 to 17). 27 tumor types were represented by a single patient.

Tumor-type strata are descriptive only. No stratum was adequately powered for formal comparison and no inference should be drawn from between-stratum differences.

Table S4. Laboratory turnaround time (sample receipt to issue of final report)

Cohort	Modality	Reports with dates (n)	Median (days)	IQR (days)	Range (days)
Matched evaluable (n=123)	Plasma CGP	115	7	6 to 8	4 to 28
Matched evaluable (n=123)	Tissue CGP	111	13	8 to 16	5 to 78
All paired sets (n=158)	Plasma CGP	147	7	6 to 8	4 to 28
All paired sets (n=158)	Tissue CGP	144	13	8 to 17	5 to 78

Turnaround time is the interval in calendar days from receipt of the sample in the testing laboratory to issue of the final report. It excludes the pre-analytical interval from biopsy or phlebotomy to receipt, and the interval from report issue to clinical review.

Table S5. Per-pair analytic dataset (de-identified, all 158 paired sample sets)

Patient	Tumor type	Tissue report status	Somatic alteration, tissue	Somatic alteration, plasma	Technically successful (both)	Informative (both)	In matched evaluable cohort	Clinically relevant, tissue	Clinically relevant, plasma	TAT tissue (d)	TAT plasma (d)
P1	Esophageal squamous cell carcinoma	FINAL	Yes	Yes	Yes	Yes	Yes	No	No	25	7
P2	Prostate adenocarcinoma	FINAL	Yes	Yes	Yes	Yes	Yes	No	Yes	13	7
P3	Cancer, Other	FINAL	Yes	Yes	Yes	Yes	Yes	No	No	8	6
P4	Breast Carcinoma	FINAL	Yes	Yes	Yes	Yes	Yes	Yes	Yes	16	5
P5	Lung adenocarcinoma	FINAL	Yes	Yes	Yes	Yes	Yes	No	No	14	5
P6	Pancreatic neuroendocrine tumor	FINAL	Yes	Yes	Yes	Yes	Yes	No	No	8	6

Patient	Tumor type	Tissue report status	Somatic alteration, tissue	Somatic alteration, plasma	Technically successful (both)	Informative (both)	In matched evaluable cohort	Clinically relevant, tissue	Clinically relevant, plasma	TAT tissue (d)	TAT plasma (d)
P7	Ovarian carcinoma	FINAL	Yes	Yes	Yes	Yes	Yes	No	No	10	5
P8	Endometrial carcinoma	FINAL	Yes	Yes	Yes	Yes	Yes	No	No	9	6
P9	Cholangiocarcinoma	FINAL	Yes	Yes	Yes	Yes	Yes	Yes	Yes	16	6
P10	Breast Carcinoma	FINAL	Yes	Yes	Yes	Yes	Yes	Yes	Yes	6	6
P11	Breast Carcinoma	FINAL	Yes	Yes	Yes	Yes	Yes	Yes	Yes	12	13
P12	Lung adenocarcinoma	FINAL	Yes	Yes	Yes	Yes	Yes	No	No	7	6
P13	Neuroendocrine carcinoma	FINAL	Yes	Yes	Yes	Yes	Yes	No	No	17	6
P14	Colorectal adenocarcinoma	FINAL	Yes	Yes	Yes	Yes	Yes	No	Yes	10	8
P15	Endometrial carcinoma	FINAL	Yes	Yes	Yes	Yes	Yes	No	No	32	5
P16	Renal Cell Carcinoma	FINAL	Yes	Yes	Yes	Yes	Yes	No	No	15	6
P17	Lung adenocarcinoma	FINAL	Yes	Yes	Yes	Yes	Yes	Yes	Yes	16	8
P18	Pancreatic ductal adenocarcinoma	FINAL	Yes	Yes	Yes	Yes	Yes	No	No	5	9
P19	Colorectal adenocarcinoma	FINAL	Yes	Yes	Yes	Yes	Yes	No	No	9	5
P20	Breast Carcinoma	FINAL	Yes	Yes	Yes	Yes	Yes	No	No	26	8
P21	Endometrial carcinoma	CORRECTED	Yes	Yes	Yes	Yes	Yes	No	No	16	22
P22	Cholangiocarcinoma	FINAL	Yes	Yes	Yes	Yes	Yes	No	No	11	6
P23	Prostate adenocarcinoma	FINAL	Yes	Yes	Yes	Yes	Yes	No	Yes	8	12
P24	Gallbladder carcinoma	FINAL	Yes	Yes	Yes	Yes	Yes	Yes	No	15	6
P25	Pancreatic ductal adenocarcinoma	FINAL	Yes	Yes	Yes	Yes	Yes	No	No	14	6
P26	Neuroendocrine carcinoma	FINAL	Yes	Yes	Yes	Yes	Yes	Yes	Yes	11	12
P27	Pancreatic ductal adenocarcinoma	FINAL	Yes	Yes	Yes	Yes	Yes	No	No	12	6
P28	Pancreatic ductal adenocarcinoma	FINAL	Yes	Yes	Yes	Yes	Yes	Yes	Yes	33	13
P29	Colorectal adenocarcinoma	FINAL	Yes	Yes	Yes	Yes	Yes	No	No	8	7
P30	Breast Carcinoma	FINAL	Yes	Yes	Yes	Yes	Yes	Yes	No	7	13
P31	Gastric adenocarcinoma	FINAL	Yes	Yes	Yes	Yes	Yes	No	No	18	5
P32	Breast Carcinoma	FINAL	Yes	Yes	Yes	Yes	Yes	No	No	14	7
P33	Prostate adenocarcinoma	FINAL	Yes	Yes	Yes	Yes	Yes	No	Yes	9	5
P34	Lung adenocarcinoma	FINAL	Yes	Yes	Yes	Yes	Yes	No	No	16	7
P35	Gastric adenocarcinoma	FINAL	Yes	Yes	Yes	Yes	Yes	No	No	8	6
P36	Neuroendocrine carcinoma	FINAL	Yes	Yes	Yes	Yes	Yes	No	No	22	7
P37	Pancreatic ductal adenocarcinoma	FINAL	Yes	Yes	Yes	Yes	Yes	No	No	15	7
P38	Lung adenocarcinoma	FINAL	Yes	Yes	Yes	Yes	Yes	Yes	Yes	6	6
P39	Urothelial carcinoma	AMENDED	Yes	Yes	Yes	Yes	Yes	No	No	19	6
P40	Lung adenocarcinoma	FINAL	Yes	Yes	Yes	Yes	Yes	Yes	No	20	4
P41	Breast Carcinoma	FINAL	Yes	Yes	Yes	Yes	Yes	No	No	21	6
P42	Other	FINAL	Yes	Yes	Yes	Yes	Yes	No	No	19	7

Patient	Tumor type	Tissue report status	Somatic alteration, tissue	Somatic alteration, plasma	Technically successful (both)	Informative (both)	In matched evaluable cohort	Clinically relevant, tissue	Clinically relevant, plasma	TAT tissue (d)	TAT plasma (d)
P43	Breast Carcinoma	FINAL	Yes	Yes	Yes	Yes	Yes	No	No	8	6
P44	Ovarian carcinoma	FINAL	Yes	Yes	Yes	Yes	Yes	No	No	8	7
P45	Lung squamous cell carcinoma	FINAL	Yes	Yes	Yes	Yes	Yes	Yes	No	9	6
P46	Breast Carcinoma	FINAL	Yes	Yes	Yes	Yes	Yes	No	No	8	6
P47	Hepatocellular carcinoma	FINAL	Yes	Yes	Yes	Yes	Yes	No	No	22	7
P48	Cholangiocarcinoma	FINAL	Yes	Yes	Yes	Yes	Yes	No	Yes	15	11
P49	Carcinoma of unknown primary (CUP)	FINAL	Yes	Yes	Yes	Yes	Yes	No	No	8	11
P50	Colorectal adenocarcinoma	FINAL	Yes	Yes	Yes	Yes	Yes	No	Yes	10	7
P51	Other	AMENDED	Yes	Yes	Yes	Yes	Yes	Yes	Yes	23	28
P52	Lung adenocarcinoma	FINAL	Yes	Yes	Yes	Yes	Yes	Yes	Yes	6	6
P53	Hepatocellular carcinoma	FINAL	Yes	Yes	Yes	Yes	Yes	No	No	7	6
P54	Lung adenocarcinoma	AMENDED	Yes	Yes	Yes	Yes	Yes	No	No	14	10
P55	Follicular thyroid carcinoma	FINAL	Yes	Yes	Yes	Yes	Yes	No	Yes	8	5
P56	Pancreatic ductal adenocarcinoma	FINAL	Yes	Yes	Yes	Yes	Yes	No	No	7	6
P57	Neuroendocrine carcinoma	FINAL	Yes	Yes	Yes	Yes	Yes	No	Yes	9	5
P58	Pancreatic ductal adenocarcinoma	FINAL	Yes	Yes	Yes	Yes	Yes	No	No	10	5
P59	Duodenal Adenocarcinoma	FINAL	Yes	Yes	Yes	Yes	Yes	Yes	No	11	14
P60	Renal Cell Carcinoma	FINAL	Yes	Yes	Yes	Yes	Yes	No	No	7	14
P61	Spindle cell carcinoma	FINAL	Yes	Yes	Yes	Yes	Yes	No	No	7	7
P62	Colorectal adenocarcinoma	FINAL	Yes	Yes	Yes	Yes	Yes	No	No	13	5
P63	Lung adenocarcinoma	FINAL	Yes	Yes	Yes	Yes	Yes	Yes	Yes	18	5
P64	Bladder urothelial (transitional cell) carcinoma	FINAL	Yes	Yes	Yes	Yes	Yes	Yes	Yes	9	6
P65	Lung adenocarcinoma	FINAL	Yes	Yes	Yes	Yes	Yes	No	No	8	6
P66	Pancreatic ductal adenocarcinoma	FINAL	Yes	Yes	Yes	Yes	Yes	No	No	19	9
P67	Sarcoma	FINAL	Yes	Yes	Yes	Yes	Yes	No	No	13	7
P68	Ewing sarcoma	FINAL	Yes	Yes	Yes	Yes	Yes	No	No	11	7
P69	Hepatocellular carcinoma	FINAL	Yes	Yes	Yes	Yes	Yes	No	No	15	13
P70	Glioblastoma	FINAL	Yes	Yes	Yes	Yes	Yes	No	No	22	5
P71	Thyroid carcinoma	FINAL	Yes	Yes	Yes	Yes	Yes	Yes	Yes	8	6
P72	Endometrial carcinoma	FINAL	Yes	Yes	Yes	Yes	Yes	No	No	12	11
P73	Duodenal Adenocarcinoma	FINAL	Yes	Yes	Yes	Yes	Yes	No	No	11	10
P74	Oral squamous cell carcinoma	FINAL	Yes	Yes	Yes	Yes	Yes	No	No	42	7
P75	Other	FINAL	Yes	Yes	Yes	Yes	Yes	No	No	13	7
P76	Ewing sarcoma	FINAL	Yes	Yes	Yes	Yes	Yes	No	No	12	11
P77	Lung adenocarcinoma	FINAL	Yes	Yes	Yes	Yes	Yes	Yes	Yes	13	7
P78	Cervical adenocarcinoma	FINAL	Yes	Yes	Yes	Yes	Yes	No	No	9	6

Patient	Tumor type	Tissue report status	Somatic alteration, tissue	Somatic alteration, plasma	Technically successful (both)	Informative (both)	In matched evaluable cohort	Clinically relevant, tissue	Clinically relevant, plasma	TAT tissue (d)	TAT plasma (d)
P79	Mesothelioma	FINAL	Yes	Yes	Yes	Yes	Yes	No	No	13	11
P80	Other	FINAL	Yes	Yes	Yes	Yes	Yes	No	No	8	7
P81	Colorectal adenocarcinoma	FINAL	Yes	Yes	Yes	Yes	Yes	No	No	8	4
P82	Renal Cell Carcinoma	FINAL	Yes	Yes	Yes	Yes	Yes	Yes	Yes	9	6
P83	Other	FINAL	Yes	Yes	Yes	Yes	Yes	No	No	78	7
P84	Colorectal adenocarcinoma	FINAL	Yes	Yes	Yes	Yes	Yes	Yes	Yes	16	6
P85	Adenoid cystic carcinoma	FINAL	Yes	Yes	Yes	Yes	Yes	No	No	17	6
P86	Urothelial carcinoma	FINAL	Yes	Yes	Yes	Yes	Yes	Yes	No	13	7
P87	Breast Carcinoma	FINAL	Yes	Yes	Yes	Yes	Yes	No	No	13	7
P88	Gemistocytic astrocytoma	FINAL	Yes	Yes	Yes	Yes	Yes	No	No	8	4
P89	Squamous cell carcinoma of the tongue	AMENDED	Yes	Yes	Yes	Yes	Yes	No	Yes	12	12
P90	Other	FINAL	Yes	Yes	Yes	Yes	Yes	No	No	14	6
P91	Glioblastoma	FINAL	Yes	Yes	Yes	Yes	Yes	No	No	8	7
P92	Pancreatic ductal adenocarcinoma	FINAL	Yes	Yes	Yes	Yes	Yes	Yes	Yes	16	8
P93	Lung adenocarcinoma	FINAL	Yes	Yes	Yes	Yes	Yes	No	Yes	19	6
P94	Colorectal adenocarcinoma	FINAL	Yes	Yes	Yes	Yes	Yes	Yes	No	7	6
P95	Sarcoma	FINAL	Yes	Yes	Yes	Yes	Yes	No	No	38	8
P96	Non-small cell lung carcinoma, NOS	FINAL	Yes	Yes	Yes	Yes	Yes	No	Yes	20	8
P97	Colorectal adenocarcinoma	FINAL	Yes	Yes	Yes	Yes	Yes	No	Yes	11	8
P98	Other	FINAL	Yes	Yes	Yes	Yes	Yes	No	No	13	4
P99	Pancreatic adenocarcinoma	FINAL	Yes	Yes	Yes	Yes	Yes	No	No	18	11
P100	Breast Carcinoma	FINAL	Yes	Yes	Yes	Yes	Yes	No	No	15	13
P101	Squamous cell carcinoma	FINAL	Yes	Yes	Yes	Yes	Yes	No	No	8	7
P102	Hepatocellular carcinoma	FINAL	Yes	Yes	Yes	Yes	Yes	Yes	Yes	8	6
P103	Colorectal adenocarcinoma	FINAL	Yes	Yes	Yes	Yes	Yes	Yes	No	15	5
P104	Lung adenocarcinoma	FINAL	Yes	Yes	Yes	Yes	Yes	No	No	8	6
P105	Squamous cell carcinoma	FINAL	Yes	Yes	Yes	Yes	Yes	No	No	11	5
P106	Glioblastoma	FINAL	Yes	Yes	Yes	Yes	Yes	No	No	11	4
P107	Lung adenocarcinoma	FINAL	Yes	Yes	Yes	Yes	Yes	Yes	Yes	9	4
P108	Ovarian carcinoma	FINAL	Yes	Yes	Yes	Yes	Yes	No	Yes		7
P109	Colorectal adenocarcinoma	FINAL	Yes	Yes	Yes	Yes	Yes	Yes	Yes		7
P110	Glioblastoma	FINAL	Yes	Yes	Yes	Yes	Yes	No	No		
P111	Diffuse midline glioma	FINAL	Yes	Yes	Yes	Yes	Yes	No	No		
P112	GIST (Gastrointestinal stromal tumor)	FINAL	Yes	Yes	Yes	Yes	Yes	Yes	Yes	10	5
P113	Carcinoma of unknown primary (CUP)	FINAL	Yes	Yes	Yes	Yes	Yes	No	No		
P114	Lung adenocarcinoma	FINAL	Yes	Yes	Yes	Yes	Yes	Yes	No	9	7

Patient	Tumor type	Tissue report status	Somatic alteration, tissue	Somatic alteration, plasma	Technically successful (both)	Informative (both)	In matched evaluable cohort	Clinically relevant, tissue	Clinically relevant, plasma	TAT tissue (d)	TAT plasma (d)
P115	Liposarcoma	FINAL	Yes	Yes	Yes	Yes	Yes	No	No	7	7
P116	Pancreatic ductal adenocarcinoma	FINAL	Yes	Yes	Yes	Yes	Yes	No	No		
P117	Glioblastoma	FINAL	Yes	Yes	Yes	Yes	Yes	No	No		10
P118	Meningioma	FINAL	Yes	Yes	Yes	Yes	Yes	No	No		9
P119	Ovarian carcinoma	FINAL	Yes	Yes	Yes	Yes	Yes	No	No		
P120	Esophageal squamous cell carcinoma	FINAL	Yes	Yes	Yes	Yes	Yes	Yes	No		5
P121	Colorectal adenocarcinoma	FINAL	Yes	Yes	Yes	Yes	Yes	Yes	Yes		
P122	Esophageal/Gastroesophageal junction Adenocarcinoma	FINAL	Yes	Yes	Yes	Yes	Yes	Yes	No	9	
P123	Pleomorphic sarcoma	FINAL	Yes	Yes	Yes	Yes	Yes	No	No		
P124	Neuroendocrine carcinoma	FINAL	No	No	Yes	No	No	No	No	16	6
P125	Thymic carcinoma	CANCELLED	No	Yes	No	No	No	No	No	78	6
P126	Other	FINAL	Yes	No	Yes	No	No	No	No	11	8
P127	Hepatocellular carcinoma	CANCELLED	No	Yes	No	No	No	No	No	59	12
P128	Lung adenocarcinoma	FINAL	No	No	Yes	No	No	No	No	47	6
P129	Pancreatic ductal adenocarcinoma	CANCELLED	No	Yes	No	No	No	No	No	64	5
P130	Cholangiocarcinoma	FINAL	No	No	Yes	No	No	No	No	10	5
P131	Prostate adenocarcinoma	CANCELLED	No	Yes	No	No	No	No	No	45	6
P132	Ovarian carcinoma	CANCELLED	No	Yes	No	No	No	No	No	63	11
P133	Glioblastoma	FINAL	Yes	No	Yes	No	No	No	No	8	7
P134	Endometrial carcinoma	FINAL	Yes	No	Yes	No	No	No	No	13	6
P135	Esophageal/Gastroesophageal junction Adenocarcinoma	CANCELLED	No	Yes	No	No	No	No	No	63	8
P136	Sertoli-Leydig cell tumor	FINAL	Yes	No	Yes	No	No	No	No	11	5
P137	Endometrial carcinoma	CANCELLED	No	Yes	No	No	No	No	No	65	8
P138	Prostate adenocarcinoma	CANCELLED	No	Yes	No	No	No	No	Yes	27	6
P139	Squamous cell carcinoma of the tongue	CANCELLED	No	Yes	No	No	No	No	No	56	5
P140	Cervical Squamous Cell Carcinoma	FINAL	Yes	No	Yes	No	No	No	No	13	7
P141	GIST (Gastrointestinal stromal tumor)	FINAL	Yes	No	Yes	No	No	Yes	No	24	7
P142	Pancreatic ductal adenocarcinoma	CANCELLED	No	Yes	No	No	No	No	No	51	11
P143	Colorectal adenocarcinoma	CANCELLED	No	Yes	No	No	No	No	Yes	43	7
P144	Pancreatic ductal adenocarcinoma	CANCELLED	No	Yes	No	No	No	No	No	7	7
P145	Synovial sarcoma	FINAL	Yes	No	Yes	No	No	No	No	8	7
P146	Langerhans cell histiocytosis (LCH)	CANCELLED	No	No	No	No	No	No	No	15	6
P147	Lung adenocarcinoma	CANCELLED	No	Yes	No	No	No	No	Yes	29	5
P148	Ewing sarcoma	FINAL	Yes	No	Yes	No	No	No	No	7	8
P149	Carcinoma of unknown primary (CUP)	CANCELLED	No	Yes	No	No	No	No	No	18	8

Patient	Tumor type	Tissue report status	Somatic alteration, tissue	Somatic alteration, plasma	Technically successful (both)	Informative (both)	In matched evaluable cohort	Clinically relevant, tissue	Clinically relevant, plasma	TAT tissue (d)	TAT plasma (d)
P150	Pancreatic ductal adenocarcinoma	CANCELLED	No	No	No	No	No	No	No	11	6
P151	Meningioma	FINAL	No	No	Yes	No	No	No	No	8	7
P152	Sinonasal adenocarcinoma	FINAL	Yes	No	Yes	No	No	No	No	11	7
P153	Gastroesophageal junction carcinoma	FINAL	Yes	No	Yes	No	No	No	No	11	5
P154	Gliosarcoma	FINAL	Yes	No	Yes	No	No	No	No	6	6
P155	Prostate adenocarcinoma	CANCELLED	No	Yes	No	No	No	No	No	9	
P156	Pancreatic carcinoid tumor	FINAL	No	No	Yes	No	No	No	No	6	13
P157	Colorectal adenocarcinoma	CANCELLED	No	Yes	No	No	No	No	Yes		
P158	Sarcoma	CANCELLED	No	Yes	No	No	No	No	No		

Tissue report status CANCELLED denotes a technically unsuccessful test. No plasma report was technically unsuccessful.

Table S6. TMB-high and MSI-high concordance between modalities (n = 123)

Biomarker	Positive in tissue	Positive in plasma	Positive in both	Tissue only	Plasma only	Positive in either
TMB-high	12	20	5	7	15	27
MSI-high	2	2	2	0	0	2
TMB-high or MSI-high	12	21	6	6	15	27

Tissue TMB was not evaluable or not reported in 16 of 123 pairs and plasma TMB in 30 of 123; these were counted as not TMB-high.

Table S7. Patient-level detection of clinically relevant findings by tissue CGP, plasma CGP and paired testing in the exploratory non-NSCLC subgroup (n = 106).

Measure	Patients, n (%)	95% CI
Clinically relevant finding detected by either modality	35/106 (33.0%)	24.8% to 42.4%
Detected by tissue CGP	24/106 (22.6%)	15.7% to 31.5%
Detected by plasma CGP	27/106 (25.5%)	18.1% to 34.5%
Detected by both tissue and plasma	16/106 (15.1%); 16/35 (45.7% of combined-positive patients)	9.5% to 23.1%; 30.5% to 61.8%
Detected by tissue only	8/106 (7.5%); 8/35 (22.9% of combined-positive patients)	3.9% to 14.2%; 12.1% to 39.0%
Detected by plasma only	11/106 (10.4%); 11/35 (31.4% of combined-positive patients)	5.9% to 17.6%; 18.6% to 48.0%
Incremental yield over tissue alone	+11 patients; +10.4 percentage points; +45.8% relative increase	Not calculated
Incremental yield over plasma alone	+8 patients; +7.5 percentage points; +29.6% relative increase	Not calculated

A clinically relevant finding was defined as in the primary analysis. The proportion of combined-positive patients detected by each modality uses the combined-positive group as the denominator and should not be interpreted as analytic sensitivity against an independent reference standard.

Percentage points are the difference between the proportion detected by either modality and by the single modality indicated; relative increase expresses the additional patients as a proportion of those detected by that single modality.
